# Supplementary material for: Dearomatization of 3-Aminophenols for Synthesis of Spiro[chromane-3,1′-cyclohexane]-2′,4′-dien-6′-ones via Hydride Transfer Strategy-Enabled [5+1] Annulations
Source: Molecules. 2024 Feb 26;29(5):1012. doi: 10.3390/molecules29051012 (PMC10933846; doi:10.3390/molecules29051012)

Supporting Information

Dearomatization of 3-Aminophenols for Synthesis of Spiro[chromane-3,1′-cyclohexane]-2′,4′-dien-6′-ones via Hydride Transfer Strategy-Enabled [5+1] Annulations

Jia-Cheng Ge [1,2,†], Yufeng Wang [1,†], Feng-Wei Guo [1], Xiangyun Kong [1], Fangzhi Hu [1,*] and Shuai-Shuai Li [1,2,*]

[1] College of Chemistry and Pharmaceutical Sciences, Qingdao Agricultural University, Qingdao 266109, China; gejiacheng@hailir.cn (J.-C.G.); wyf1758561298@163.com (Y.W.); guofengwei2017@163.com (F.-W.G.); kongxiangyun528@126.com (X.K.)
[2] Hailir Pesticides and Chemicals Group Co., Ltd., Qingdao 266109, China
* Correspondence: ssfangzhi@126.com (F.H.); flyshuaishuai@126.com (S.-S.L.)
† These authors contributed equally to this work.

Table of Contents

1. General Information……………………………………………………………S2

2. General Procedure…………………………………………………………...S2

3. Control Experiments…………………………………………………………...S3

4. Characterization of Products…………………………………………………S4

5. In Vitro Antifungal Activities………………………………………………..S15

6. Crystal Structures and Data…………………………………….……………..S17

7. ^{1}H and ^{13}C NMR Spectra…………………………………………………S18

1. General Information

Unless otherwise noted, all reagents and solvents were purchased from the commercial sources (from Adamas-beta, Shanghai, China) and used as received. Thin layer chromatography (TLC) was used to monitor the reaction on Merck 60 F254 precoated silica gel plate (0.2 mm thickness). TLC spots were visualized by UV-light irradiation on Spectroline Model ENF-24061/F 254 nm. The products were purified by flash column chromatography (200-300 mesh silica gel) eluted with the gradient of petroleum ether and ethyl acetate. Proton nuclear magnetic resonance spectra (^{1}H NMR) were recorded on a Bruker 500 MHz or 400 MHz NMR spectrometer ($CDCl_3$, DMSO-d_6 or Methanol-d_4 solvent). The chemical shifts were reported in parts per million (ppm), downfield from $SiMe_4$ (δ 0.0) and relative to the signal of chloroform-*d* (δ 7.26, singlet), dimethyl sulfoxide-d_6 (δ 2.54, singlet) or methanol-d_4 (δ 3.31, quintuplet). Multiplicities were afforded as: s (singlet); d (doublet); t (triplet); q (quartet); dd (doublets of doublet) or m (multiplets). The number of protons for a given resonance is indicated by nH. Coupling constants were reported as a *J* value in Hz. Carbon nuclear magnetic resonance spectra (^{13}C NMR) was referenced to the appropriate residual solvent peak. High resolution mass spectral analysis (HRMS) was performed on Waters XEVO G2 Q-TOF. The *ortho*-substituted benzaldehydes were prepared according to the literature[1].

2. General Procedure

2.1 General Procedure for the Dearomative [5+1] Annulation

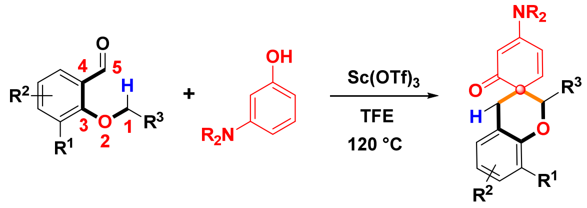

1 **2** **3**

A sealed tube was charged with *O*-alkyl *ortho*-oxybenzaldehyde **1** (0.1 mmol), phenol **2** (0.15 mmol), $Sc(OTf)_3$ (20 mol%), and TFE (1.0 mL). The mixture was stirred at 120 °C for 5 h. Upon completion of the reaction as indicated by TLC analysis, the mixture was concentrated in vacuum and the residue was directly purified by flash column chromatography on silica gel (eluent: ethyl acetate/petroleum ether, 1:3) to afford the desired spiro[chromane-3,1'-cyclohexane]-2',4'-dien-6'-ones **3a**–**u**.

2.2 Gram-scale Synthesis and Derivatization of Product 3

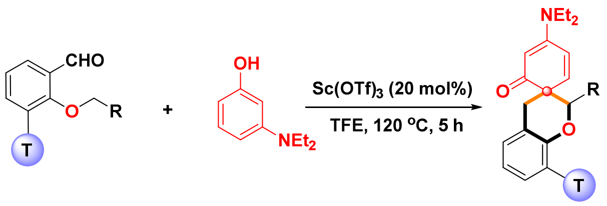

R = Ph, 1a (4 mmol, 1.07 g) **2a** (6 mmol, 0.99 g) **R = Ph, 3a**, 1.11 g, 67%
R = Vinyl, 1k (4 mmol, 0.87 g) **2a** (6 mmol, 0.99 g) **R = Vinyl, 3k**, 1.08 g, 74%

A round-bottom flask was charged with *O*-alkyl *ortho*-oxybenzaldehydes **1a** (4.0 mmol, 1.07 g) or **1k** (4.0 mmol, 0.87 g), phenol **2a** (6.0 mmol, 0.99 g), $Sc(OTf)_3$ (20 mol%), and TFE (40.0 mL). The mixture was stirred

at 120 °C for 5 h. Upon completion of the reaction as indicated by TLC analysis, the mixture was concentrated in vacuum and the residue was directly purified by flash column chromatography on silica gel (eluent: ethyl acetate/petroleum ether, 1:3) to afford the desired spiro[chromane-3,1'-cyclohexane]-2',4'-dien-6'-ones **3a** in 67% yield or **3k** in 74% yield, respectively.

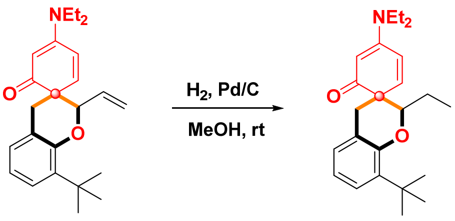

3k **4k**, 82%, dr 1:1

A reaction tube was charged with spiro[chromane-3,1'-cyclohexane]-2',4'-dien-6'-one **3k** (0.1 mmol, 36.6 mg) and 30% by wt Pd/C (10 % by wt relative to **3k**) in 1.0 mL of MeOH. The tube was equipped with a magnetic stir bar, and the suspension was sealed with a septum under an atmosphere of H_2 supplied via a balloon for 6 h. Upon completion of the reaction as indicated by TLC analysis, the suspension was filtered through a pad of Celite. The filtrate was concentrated in vacuum. The residue was directly purified by flash column chromatography on silica gel (ethyl acetate: petroleum ether, 1:3) to give the desired product **4k** in 82% yield and 1:1 diastereoselectivity.

2.3 The Procedure for Synthesis of Product 5a

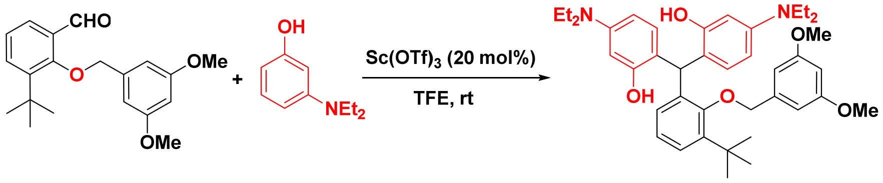

1v **2a** **5a**, 81%

A tube was charged with *O*-alkyl *ortho*-oxybenzaldehyde **1v** (0.1 mmol, 32.8 mg), phenol **2a** (0.22 mmol, 36.4 mg), Sc(OTf)3 (20 mol%), and TFE (1.0 mL). The mixture was stirred at room temperature for 5 h. Upon completion of the reaction as indicated by TLC analysis, the mixture was concentrated in vacuum and the residue was directly purified by flash column chromatography on silica gel (eluent: ethyl acetate/petroleum ether, 1:15) to afford the polyarylated methane **5a** in 81% yield.

3. Control Experiments

3.1 Investigation on the "buttressing effect"

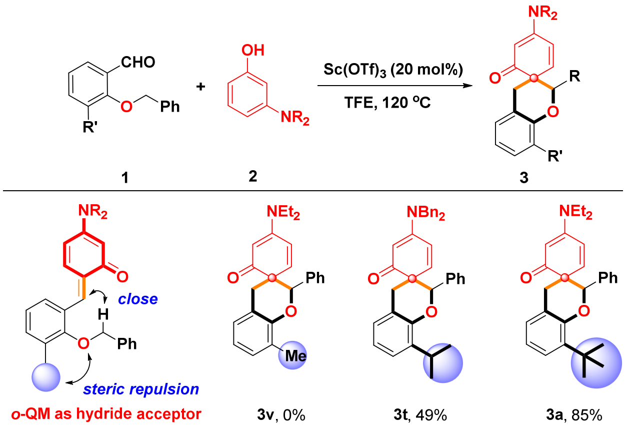

A tube was charged with *O*-alkyl *ortho*-oxybenzaldehydes **1** bearing methyl, isopropyl, or tertbutyl (0.1 mmol), phenol **2** (0.15 mmol), $Sc(OTf)_3$ (20 mol%), and TFE (1.0 mL). The mixture was stirred at 120 °C for 5 h. Upon completion of the reaction as indicated by TLC analysis, the mixture was concentrated in vacuum and the residue was directly purified by flash column chromatography on silica gel (eluent: ethyl acetate/petroleum ether, 1:3) to afford the desired spiro[chromane-3,1'-cyclohexane]-2',4'-dien-6'-ones **3v**, **3t**, or **3a**. Remarkable enhancement of the reactivity by the bulky group ortho to the alkoxy group could be clearly observed which might be due to the steric repulsion between ortho group and alkoxy group made the distance closer between hydride donor and hydride acceptor.

3.2 Investigation on the effect of hydride donors

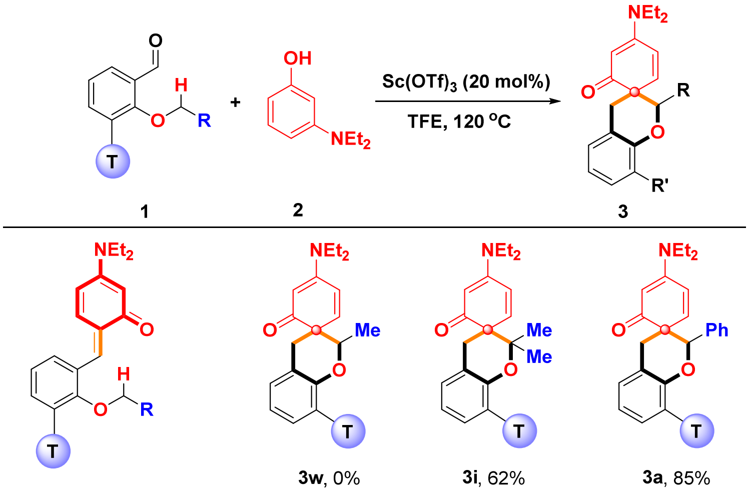

A tube was charged with *O*-ethyl, *O*-isopropyl, or *O*-benzyl *ortho*-oxybenzaldehydes **1** (0.1 mmol), phenol **2** (0.15 mmol), $Sc(OTf)_3$ (20 mol%), and TFE (1.0 mL). The mixture was stirred at 120 °C for 5 h. Upon completion of the reaction as indicated by TLC analysis, the mixture was concentrated in vacuum and the residue was directly purified by flash column chromatography on silica gel (eluent: ethyl acetate/petroleum ether, 1:3) to afford the desired spiro[chromane-3,1'-cyclohexane]-2',4'-dien-6'-ones **3w**, **3i**, or **3a**. Remarkable enhancement of the reactivity by the bulky group ortho to the alkoxy group could be clearly observed which might be due to the steric repulsion between ortho group and alkoxy group made the distance closer between hydride donor and hydride

acceptor. The examination of the α-C(sp^3)−H bond of ethyl, *iso*-propyl, and benzyl adjacent to oxygen showed the difference in the activity which might be depended on the transfer ability of the hydride donors and the stability of the cations generated upon hydride transfer.

4. Characterization of Products

8-(tert-butyl)-4'-(diethylamino)-2-phenylspiro[chromane-3,1'-cyclohexane]-2',4'-dien-6'-one (3a)

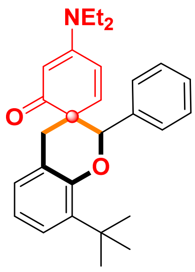

Flash column chromatography on a silica gel (ethyl acetate: petroleum ether, 1:3) afforded the product (35.3 mg, 85% yield) as a white solid.

^{1}H NMR (500 MHz, $CDCl_3$) δ 7.30 (m, 2H), 7.17 – 7.08 (m, 4H), 6.95 (d, *J* = 7.4 Hz, 1H), 6.82 (d, *J* = 7.6 Hz, 1H), 6.56 (d, *J* = 10.4 Hz, 1H), 6.26 (dd, *J* = 10.4, 2.1 Hz, 1H), 5.24 (s, 1H), 4.93 (d, *J* = 2.0 Hz, 1H), 3.85 (d, *J* = 16.5 Hz, 1H), 3.16 – 3.01 (m, 4H), 2.62 (d, *J* = 16.6 Hz, 1H), 1.30 (s, 9H), 0.93 (t, *J* = 7.0 Hz, 6H). **^{13}C NMR** (125 MHz, $CDCl_3$) δ 195.4, 156.2, 152.6, 142.2, 137.9, 137.1, 127.9, 127.6, 127.1, 124.4, 121.0, 120.4, 119.0, 97.4, 83.5, 49.9, 45.0, 36.9, 34.8, 29.9. **HRMS (ESI):** $[M+Na]^+$ calcd. for $C_{28}H_{33}NNaO_2^+$: 438.2404, found: 438.2408.

8-(tert-butyl)-4'-(diethylamino)-2-(2-fluorophenyl)spiro[chromane-3,1'-cyclohexane]-2',4'-dien-6'-one (3b)

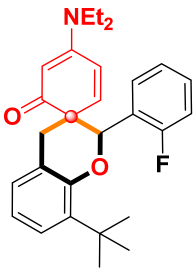

Flash column chromatography on a silica gel (ethyl acetate: petroleum ether, 1:3) afforded the product (20.4 mg, 47% yield) as a yellow solid.

^{1}H NMR (500 MHz, $CDCl_3$) δ 7.41 (t, *J* = 6.9 Hz, 1H), 7.21 (d, *J* = 6.7 Hz, 1H), 7.18 (d, *J* = 7.7 Hz, 1H), 7.02 (m, 2H), 6.99 – 6.94 (m, 1H), 6.89 (t, *J* = 7.6 Hz, 1H), 6.71 (d, *J* = 10.4 Hz, 1H), 6.43 (dd, *J* = 10.4, 1.8 Hz, 1H), 5.61 (s, 1H), 5.05 (d, *J* = 1.4 Hz, 1H), 3.97 (d, *J* = 16.6 Hz, 1H), 3.21 (d, *J* = 6.2 Hz, 4H), 2.70 (d, *J* = 16.6 Hz, 1H), 1.34 (s, 9H), 1.04 (t, *J* = 6.7 Hz, 6H). **^{13}C NMR** (125 MHz, $CDCl_3$) δ 194.5, 160.2 (d, *J* = 248.7 Hz), 156.0, 152.5, 142.2, 137.8, 129.5 (d, *J* = 3.8 Hz), 129.3 (d, *J* = 8.8 Hz), 127.9, 124.5 (d, *J* = 12.5 Hz), 124.4, 122.6 (d, *J* = 3.8 Hz), 120.8, 120.5, 119.0, 115.3, 115.2, 96.7, 77.6, 49.2, 44.9, 36.8, 34.7, 29.7. **HRMS (ESI):** $[M+Na]^+$calcd. for $C_{28}H_{32}FNNaO_2^+$: 456.2309, found: 456.2311.

8-(tert-butyl)-2-(3-chlorophenyl)-4'-(diethylamino)spiro[chromane-3,1'-cyclohexane]-2',4'-dien-6'-one (3c)

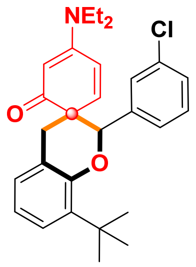

Flash column chromatography on a silica gel (ethyl acetate: petroleum ether, 1:3) afforded the product (25.6 mg, 57% yield) as a white solid.

^{1}H NMR (500 MHz, $CDCl_3$) δ 7.40 (s, 1H), 7.28 (s, 1H), 7.23 – 7.19 (m, 2H), 7.17 (t, *J* = 7.7 Hz, 1H), 7.04 (d, *J* = 7.4 Hz, 1H), 6.91 (t, *J* = 7.6 Hz, 1H), 6.62 (d, *J* = 10.4 Hz, 1H), 6.40 (dd, *J* = 10.4, 2.2 Hz, 1H), 5.28 (s, 1H), 5.05 (d, *J* = 2.1 Hz, 1H), 3.92 (d, *J* = 16.6 Hz, 1H), 3.22 (d, *J* = 6.4 Hz, 4H), 2.71 (d, *J* = 16.7 Hz, 1H), 1.39 (s, 9H), 1.05 (t, *J* = 7.0 Hz, 6H). **^{13}C NMR** (125 MHz, $CDCl_3$) δ 194.8, 156.1, 152.3, 141.7, 139.1, 137.9, 132.8, 128.4, 127.9, 127.7, 127.3, 126.1, 124.5, 120.8, 120.6, 119.3, 97.3, 82.7, 49.7, 45.0, 36.5, 34.7, 29.8. **HRMS (ESI):** $[M+Na]^+$ calcd. for $C_{28}H_{32}ClNaNO_2^+$: 472.2014, found: 472.2010.

8-(tert-butyl)-4'-(diethylamino)-2-(m-tolyl)spiro[chromane-3,1'-cyclohexane]-2',4'-dien-6'-one (3d)

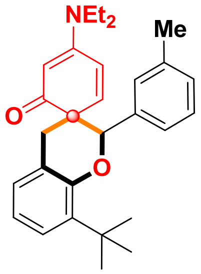

Flash column chromatography on a silica gel (ethyl acetate: petroleum ether, 1:3) afforded the product (38.2 mg, 89% yield) as a white solid.

^{1}H NMR (500 MHz, $CDCl_3$) δ 7.16 (m, 3H), 7.08 (t, *J* = 7.5 Hz, 1H), 7.00 (d, *J* = 7.3 Hz, 2H), 6.86 (t, *J* = 7.6 Hz, 1H), 6.61 (d, *J* = 10.4 Hz, 1H), 6.32 (dd, *J* = 10.4, 2.2 Hz, 1H), 5.25 (s, 1H), 4.99 (d, *J* = 2.2 Hz, 1H), 3.90 (d, *J* = 16.6 Hz, 1H), 3.28 – 3.01 (m, 4H), 2.67 (d, *J* = 16.6 Hz, 1H), 2.27 (s, 3H), 1.36 (s, 9H), 0.99 (t, *J* = 7.1 Hz, 6H). **^{13}C NMR** (125 MHz, $CDCl_3$) δ 195.2, 156.1, 152.5, 142.1, 137.7, 136.9, 136.2, 128.2, 128.2, 127.8, 126.8, 124.5, 124.3, 120.8, 120.2, 118.8, 97.3, 83.4, 49.7, 44.9, 36.8, 34.7, 29.7, 21.4. **HRMS (ESI):** $[M+Na]^+$ calcd. for $C_{29}H_{35}NNaO_2^+$: 452.2560, found: 452.2558.

8-(tert-butyl)-4'-(diethylamino)-2-(p-tolyl)spiro[chromane-3,1'-cyclohexane]-2',4'-dien-6'-one (3e)

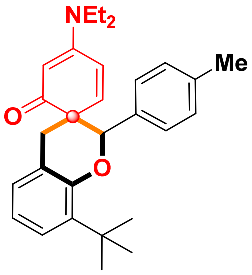

Flash column chromatography on a silica gel (ethyl acetate: petroleum ether, 1:3) afforded the product (39.1 mg, 91% yield) as a white solid.

^{1}H NMR (500 MHz, $CDCl_3$) δ 7.29 (d, *J* = 1.9 Hz, 1H), 7.27 (s, 1H), 7.22 – 7.16 (m, 1H), 7.04 (m, 3H), 6.89 (t, *J* = 7.6 Hz, 1H), 6.65 (d, *J* = 10.4 Hz, 1H), 6.36 (dd, *J* = 10.4, 2.2 Hz, 1H), 5.30 (s, 1H), 5.02 (d, *J* = 2.2 Hz, 1H), 3.93 (d, *J* = 16.6 Hz, 1H), 3.20 (d, *J* = 6.7 Hz, 4H), 2.70 (d, *J* = 16.6 Hz, 1H), 2.30 (s, 3H), 1.40 (s, 9H), 1.03 (t, *J* = 7.1 Hz, 6H). **^{13}C NMR** (125 MHz, $CDCl_3$) δ 195.3, 156.1, 152.6, 142.2, 137.7, 137.0, 134.1, 127.8, 127.6, 127.3, 124.3, 120.8, 120.2, 118.9, 97.3, 83.3, 49.8, 44.85, 36.8, 34.7, 29.7, 21.1. **HRMS (ESI):** $[M+Na]^+$ calcd. for $C_{29}H_{35}NNaO_2^+$: 452.2560, found: 452.2556.

8-(tert-butyl)-4'-(diethylamino)-2-(4-fluorophenyl)spiro[chromane-3,1'-cyclohexane]-2',4'-dien-6'-one (3f)

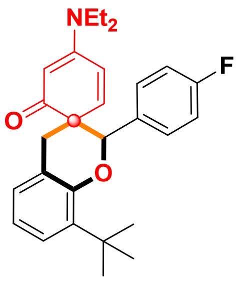

Flash column chromatography on a silica gel (ethyl acetate: petroleum ether, 1:3) afforded the product (34.6 mg, 80% yield) as a yellow solid.

^{1}H NMR (500 MHz, $CDCl_3$) δ 7.40 – 7.31 (m, 2H), 7.18 (dd, *J* = 7.9, 1.7 Hz, 1H), 7.02 (dd, *J* = 7.5, 1.6 Hz, 1H), 6.95 – 6.85 (m, 3H), 6.62 (d, *J* = 10.5 Hz, 1H), 6.36 (dd, *J* = 10.5, 2.3 Hz, 1H), 5.30 (s, 1H), 5.02 (s, 1H), 3.89 (dt, *J* = 16.5, 1.1 Hz, 1H), 3.20 (q, *J* = 7.2 Hz, 4H), 2.69 (d, *J* = 16.6 Hz, 1H), 1.37 (s, 9H), 1.03 (t, *J* = 7.1 Hz, 6H). **^{13}C NMR** (100 MHz, $CDCl_3$) δ 195.2, 162.3 (d, *J* = 244.0 Hz, 1H), 156.2, 152.5, 142.1, 137.9, 129.2 (d, *J* = 8.0 Hz, 1H), 127.9, 124.5, 120.9, 120.6, 119.2, 113.9 (d, *J* = 21.0 Hz, 1H), 97.3, 82.8, 49.8, 45.0, 36.9, 34.8, 29.8. **HRMS (ESI):** $[M+Na]^+$calcd. for $C_{28}H_{32}FNNaO_2^+$: 456.2309, found: 456.2311.

2-(4-bromophenyl)-8-(tert-butyl)-4'-(diethylamino)spiro[chromane-3,1'-cyclohexane]-2',4'-dien-6'-one (3g)

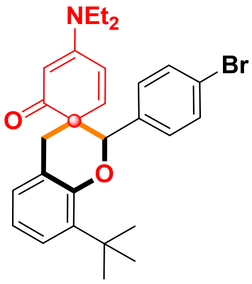

Flash column chromatography on a silica gel (ethyl acetate: petroleum ether, 1:3) afforded the product (25.6 mg, 52% yield) as a white solid.

^{1}H NMR (500 MHz, $CDCl_3$) δ 7.28 (d, *J* = 8.5 Hz, 2H), 7.20 (s, 1H), 7.18 (s, 1H), 7.11 (d, *J* = 7.4 Hz, 1H), 6.95 (d, *J* = 7.4 Hz, 1H), 6.82 (t, *J* = 7.6 Hz, 1H), 6.53 (d, *J* = 10.4 Hz, 1H), 6.28 (dd, *J* = 10.4, 2.2 Hz, 1H), 5.20 (s, 1H), 4.95 (d, *J* = 2.1 Hz, 1H), 3.82 (d, *J* = 16.6 Hz, 1H), 3.14 (d, *J* = 5.4 Hz, 4H), 2.62 (d, *J* = 16.7 Hz, 1H), 1.29 (s, 9H), 0.97 (t, *J* = 7.1 Hz, 6H). **^{13}C NMR** (125 MHz, $CDCl_3$) δ 195.7, 156.9, 153.1, 142.6, 138.6, 137.0, 131.0,

130.0, 128.6, 125.3, 122.3, 121.6, 121.4, 120.0, 98.1, 83.5, 50.5, 45.8, 37.5, 35.5, 30.6. **HRMS (ESI):** $[M+Na]^+$ calcd. for $C_{28}H_{32}BrNNaO_2^+$: 516.1509, found: 516.1496.

8-(tert-butyl)-4'-(diethylamino)-2-(naphthalen-2-yl)spiro[chromane-3,1'-cyclohexane]-2',4'-dien-6'-one (3h)

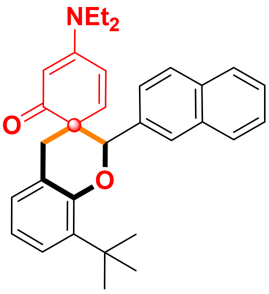

Flash column chromatography on a silica gel (ethyl acetate: petroleum ether, 1:3) afforded the product (41.8 mg, 90% yield) as a white solid.

^{1}H NMR (500 MHz, $CDCl_3$) δ 7.86 (s, 1H), 7.84 – 7.80 (m, 1H), 7.80 – 7.76 (m, 1H), 7.70 (d, *J* = 8.5 Hz, 1H), 7.55 (dd, *J* = 8.5, 1.7 Hz, 1H), 7.45 – 7.41 (m, 2H), 7.22 (d, *J* = 7.0 Hz, 1H), 7.06 (d, *J* = 7.0 Hz, 1H), 6.92 (t, *J* = 7.6 Hz, 1H), 6.74 (d, *J* = 10.4 Hz, 1H), 6.34 (dd, *J* = 10.5, 2.2 Hz, 1H), 5.50 (s, 1H), 4.98 (d, *J* = 2.2 Hz, 1H), 3.98 (d, *J* = 16.6 Hz, 1H), 3.05 (s, 4H), 2.75 (d, *J* = 16.6 Hz, 1H), 1.41 (s, 9H), 0.83 (s, 6H). **^{13}C NMR** (125 MHz, $CDCl_3$) δ 195.2, 156.1, 152.6, 142.1, 137.9, 134.8, 133.0, 132.5, 128.3, 127.9, 127.3, 126.6, 126.4, 125.8, 125.6, 125.5, 124.4, 120.9, 120.4, 119.1, 97.2, 83.43, 49.94, 44.8, 37.0, 34.8, 29.8. **HRMS (ESI):** $[M+Na]^+$ calcd. for $C_{32}H_{35}NNaO_2^+$: 488.2560, found: 488.2550.

8-(tert-butyl)-4'-(diethylamino)-2,2-dimethylspiro[chromane-3,1'-cyclohexane]-2',4'-dien-6'-one (3i)

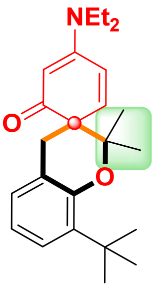

Flash column chromatography on a silica gel (ethyl acetate: petroleum ether, 1:3) afforded the product (22.8 mg, 62% yield) as a yellow oil.

^{1}H NMR (500 MHz, $CDCl_3$) δ 7.15 (dd, *J* = 7.8, 1.7 Hz, 1H), 7.00 – 6.92 (m, 1H), 6.82 (t, *J* = 7.6 Hz, 1H), 6.48 (d, *J* = 10.5 Hz, 1H), 6.42 (dd, *J* = 10.5, 2.3 Hz, 1H), 5.31 (d, *J* = 2.3 Hz, 1H), 3.69 – 3.58 (m, 1H), 3.39 (q, *J* = 7.1 Hz, 4H), 2.46 (d, *J* = 16.9 Hz, 1H), 1.47 (s, 3H), 1.39 (s, 9H), 1.33 (s, 3H), 1.22 (t, *J* = 7.1 Hz, 6H). **^{13}C NMR** (125 MHz, $CDCl_3$) δ 195.9, 156.4, 151.1, 146.1, 138.1, 127.7, 124.3, 120.9, 119.6, 117.8, 97.4, 79.1, 77.2, 50.2, 44.9, 34.8, 33.8, 30.4, 29.7, 24.6, 21.6. **HRMS (ESI):** $[M+Na]^+$ calcd. for $C_{24}H_{33}NNaO_2^+$: 390.2404, found: 390.2409.

8-(tert-butyl)-4'-(diethylamino)-2-methyl-2-phenylspiro[chromane-3,1'-cyclohexane]-2',4'-dien-6'-one (3j)

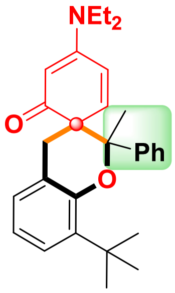

Flash column chromatography on a silica gel (ethyl acetate: petroleum ether, 1:3) afforded the product (30.0 mg, 70% yield) as a yellow oil.

^{1}H NMR (500 MHz, $CDCl_3$) δ 7.54 – 7.46 (m, 2H), 7.23 (dd, *J* = 7.9, 1.7 Hz, 1H), 7.20 – 7.12 (m, 3H), 7.03 (dd, *J* = 7.5, 1.6 Hz, 1H), 6.91 (t, *J* = 7.6 Hz, 1H), 6.50 (d, *J* = 10.5 Hz, 1H), 6.08 (dd, *J* = 10.5, 2.3 Hz, 1H), 5.08 (d, *J* = 2.3 Hz, 1H), 3.83 (d, *J* = 17.1 Hz, 1H), 3.09 (d, *J* = 7.4 Hz, 4H), 2.58 (d, *J* = 17.2 Hz, 1H), 1.86 (s, 3H), 1.45 (s, 9H), 0.93 (t, *J* = 7.1 Hz, 6H). **^{13}C NMR** (125 MHz, $CDCl_3$) δ 195.2, 156.1, 150.7, 144.6, 142.3, 138.9, 127.9, 126.7, 126.7, 126.4, 124.7, 121.3, 120.4, 118.1, 98.5, 83.3, 50.9, 44.9, 34.9, 33.4, 30.2, 29.7, 20.1. **HRMS (ESI):** $[M+Na]^+$ calcd. for $C_{29}H_{35}NNaO_2^+$: 452.2560, found: 452.2563.

8-(tert-butyl)-4'-(diethylamino)-2-vinylspiro[chromane-3,1'-cyclohexane]-2',4'-dien-6'-one (3k)

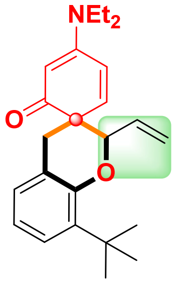

Flash column chromatography on a silica gel (ethyl acetate: petroleum ether, 1:5) afforded the product (32.9 mg, 90% yield) as a white solid.

^{1}H NMR (500 MHz, $CDCl_3$) δ 7.16 (d, *J* = 7.5 Hz, 1H), 6.95 (d, *J* = 7.3 Hz, 1H), 6.84 (t, *J* = 7.6 Hz, 1H), 6.58 – 6.42 (m, 2H), 5.86 – 5.71 (m, 1H), 5.51 (d, *J* = 17.2 Hz, 1H), 5.33 (d, *J* = 2.0 Hz, 1H), 5.19 (d, *J* = 10.8 Hz, 1H), 4.81 (d, *J* = 5.2 Hz, 1H), 3.67 (d, *J* = 16.4 Hz, 1H), 3.39 (dd, *J* = 14.1, 7.0 Hz, 4H), 2.61 (d, *J* = 16.4 Hz, 1H), 1.42 (s, 9H), 1.23 (t, *J* = 7.1 Hz, 6H). **^{13}C NMR** (125 MHz, $CDCl_3$) δ 195.9, 156.6, 152.0, 143.1, 137.6, 133.3, 127.6, 124.4, 120.8, 120.1, 118.3, 117.2, 96.6, 80.8, 48.1, 44.9, 37.6, 34.7, 29.6. **HRMS (ESI):** $[M+Na]^+$ calcd. for $C_{24}H_{31}NNaO_2^+$: 388.2247, found: 388.2244.

8-(tert-butyl)-4'-(dimethylamino)-2-phenylspiro[chromane-3,1'-cyclohexane]-2',4'-dien-6'-one (3l)

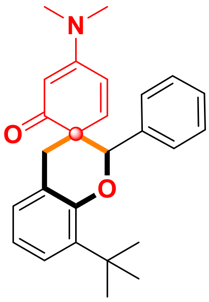

Flash column chromatography on a silica gel (ethyl acetate: petroleum ether, 1:3) afforded the product (34.8 mg, 90% yield) as a white solid.

^{1}H NMR (500 MHz, $CDCl_3$) δ 7.31 (dd, J = 7.3, 2.4 Hz, 2H), 7.18 – 7.13 (m, 3H), 7.12 – 7.07 (m, 1H), 6.91 (d, J = 7.4 Hz, 1H), 6.79 (t, J = 7.6 Hz, 1H), 6.61 (d, J = 10.5 Hz, 1H), 6.32 (dd, J = 10.4, 2.3 Hz, 1H), 5.30 (s, 1H), 4.92 (d, J = 2.3 Hz, 1H), 3.77 (d, J = 16.5 Hz, 1H), 2.79 (s, 6H), 2.59 (d, J = 16.5 Hz, 1H), 1.30 (s, 9H). **^{13}C NMR** (125 MHz, $CDCl_3$) δ 195.9, 158.1, 152.6, 142.4, 137.9, 137.5, 127.7, 127.7, 127.6, 127.3, 124.5, 120.9, 120.4, 118.7, 97.6, 82.9, 50.0, 39.9, 38.3, 34.8, 29.9. **HRMS (ESI):** $[M+Na]^+$ calcd. for $C_{26}H_{29}NNaO_2^+$: 410.2091, found: 410.2094.

8-(tert-butyl)-2-phenyl-4'-(pyrrolidin-1-yl)spiro[chromane-3,1'-cyclohexane]-2',4'-dien-6'-one (3m)

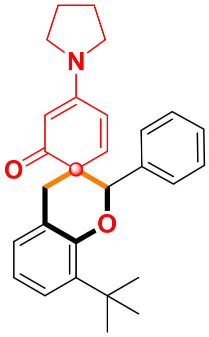

Flash column chromatography on a silica gel (ethyl acetate: petroleum ether, 1:3) afforded the product (17.7 mg, 43% yield) as a white solid.

^{1}H NMR (500 MHz, $CDCl_3$) δ 7.34 (dd, J = 7.5, 1.7 Hz, 2H), 7.20 – 7.15 (m, 3H), 7.11 (d, J = 7.5 Hz, 1H), 6.92 (d, J = 7.4 Hz, 1H), 6.80 (t, J = 7.6 Hz, 1H), 6.63 (d, J = 10.3 Hz, 1H), 6.23 (dd, J = 10.3, 1.9 Hz, 1H), 5.35 (s, 1H), 4.87 (d, J = 1.8 Hz, 1H), 3.78 (d, J = 16.5 Hz, 1H), 3.32 (m, 2H), 3.03 (m, 2H), 2.60 (d, J = 16.5 Hz, 1H), 1.82 (s, 4H), 1.31 (s, 9H). **^{13}C NMR** (125 MHz, $CDCl_3$) δ 195.3, 155.7, 152.6, 142.7, 137.9, 137.7, 127.7, 127.7, 127.6, 127.3, 124.5, 121.0, 120.3, 119.9, 97.3, 82.8, 50.3, 48.0, 47.8, 38.6, 34.8, 29.9, 25.3, 24.6. **HRMS (ESI):** $[M+Na]^+$ calcd. for $C_{28}H_{31}NNaO_2^+$: 436.2247, found: 436.2251.

(2R)-8-(tert-butyl)-2-phenyl-4'-(piperidin-1-yl)spiro[chromane-3,1'-cyclohexane]-2',4'-dien-6'-one (3n)

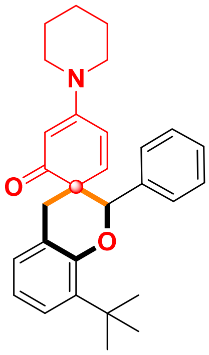

Flash column chromatography on a silica gel (ethyl acetate: petroleum ether, 1:5) afforded the product (27.3 mg, 64% yield) as a white solid.

^{1}H NMR (500 MHz, $CDCl_3$) δ 7.42 – 7.36 (m, 2H), 7.24 (m, 3H), 7.19 (d, J = 7.7 Hz, 1H), 7.03 (d, J = 7.4 Hz, 1H), 6.89 (t, J = 7.6 Hz, 1H), 6.62 (d, J = 10.4 Hz, 1H), 6.40 (d, J = 10.4 Hz, 1H), 5.32 (s, 1H), 5.13 (s, 1H), 3.91 (d, J = 16.5 Hz, 1H), 3.24 (t, J = 5.2 Hz, 4H), 2.71 (d, J = 16.6 Hz, 1H), 1.63 – 1.54 (m, 2H), 1.44 – 1.32 (m, 13H). **^{13}C NMR** (125 MHz, $CDCl_3$) δ 196.2, 157.4, 152.6, 141.6, 137.8, 137.1, 127.8, 127.6, 127.5, 127.1, 124.4, 120.8, 120.4, 119.1, 99.1, 83.4, 49.9, 48.2, 36.9, 34.7, 29.8, 25.2, 24.3. **HRMS (ESI):** calcd. for $C_{29}H_{33}NO_2$ $[M+Na]^+$: 450.2404, found: 450.2404. **HRMS (ESI):** $[M+Na]^+$ calcd. for $C_{29}H_{33}NNaO_2^+$: 450.2404, found: 450.2408.

4'-(azepan-1-yl)-8-(tert-butyl)-2-phenylspiro[chromane-3,1'-cyclohexane]-2',4'-dien-6'-one (3o)

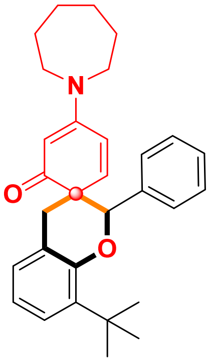

Flash column chromatography on a silica gel (ethyl acetate: petroleum ether, 1:3) afforded the product (26.9 mg, 61% yield) as a white solid.

^{1}H NMR (500 MHz, $CDCl_3$) δ 7.46 – 7.37 (m, 2H), 7.28 – 7.16 (m, 4H), 7.05 (dd, J = 7.5, 1.5 Hz, 1H), 6.91 (t, J = 7.6 Hz, 1H), 6.65 (d, J = 10.4 Hz, 1H), 6.41 (dd, J = 10.5, 2.3 Hz, 1H), 5.34 (s, 1H), 5.07 (d, J = 2.2 Hz, 1H), 3.95 (d, J = 16.5 Hz, 1H), 3.67 – 3.07 (m, 4H), 2.71 (d, J = 16.6 Hz, 1H), 1.58 (s, 4H), 1.40 (s, 9H), 1.28 (s, 4H). **^{13}C NMR** (125 MHz, $CDCl_3$) δ 195.5, 157.2, 152.6, 142.2, 137.9, 137.2, 127.9, 127.7, 127.6, 127.1, 124.4, 120.9, 120.4, 118.8, 97.6, 83.5, 49.9, 36.9, 34.8, 29.9. **HRMS (ESI):** $[M+Na]^+$ calcd. for $C_{30}H_{35}NNaO_2^+$: 464.2560, found: 464.2566.

8-(tert-butyl)-4'-(dibenzylamino)-2-phenylspiro[chromane-3,1'-cyclohexane]-2',4'-dien-6'-one (3p)

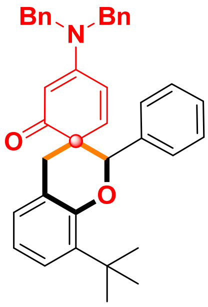

Flash column chromatography on a silica gel (ethyl acetate: petroleum ether, 1:3) afforded the product (38.3 mg, 71% yield) as a white solid.

^{1}H NMR (500 MHz, $CDCl_3$) δ 7.42 (m, 3H), 7.37 – 7.27 (m, 8H), 7.21 (d, *J* = 7.4 Hz, 1H), 7.06 (d, *J* = 7.3 Hz, 1H), 6.91 (m, 5H), 6.67 (d, *J* = 10.4 Hz, 1H), 6.43 (dd, *J* = 10.4, 2.1 Hz, 1H), 5.32 (s, 1H), 5.24 (d, *J* = 2.1 Hz, 1H), 4.41 (s, 4H), 3.96 (d, *J* = 16.6 Hz, 1H), 2.77 (d, *J* = 16.7 Hz, 1H), 1.40 (s, 9H). **^{13}C NMR** (100 MHz, $CDCl_3$) δ 196.4, 158.3, 152.6, 142.9, 137.9, 137.1, 128.9, 127.9, 127.7, 127.6, 127.3, 126.3, 124.5, 120.7, 120.6, 119.2, 98.7, 83.8, 50.4, 36.4, 34.8, 29.9. **HRMS (ESI):** $[M+Na]^+$ calcd. for $C_{38}H_{37}NNaO_2^+$: 562.2717, found: 562.2724.

8-(tert-butyl)-4'-(diallylamino)-2-phenylspiro[chromane-3,1'-cyclohexane]-2',4'-dien-6'-one (3q)

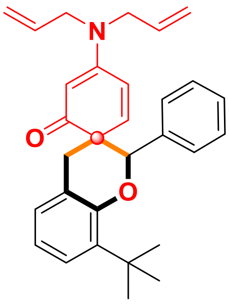

Flash column chromatography on a silica gel (ethyl acetate: petroleum ether, 1:3) afforded the product (36.4 mg, 83% yield) as a yellow oil.

^{1}H NMR (500 MHz, $CDCl_3$) δ 7.36 (m, 2H), 7.23 (m, 3H), 7.19 (d, *J* = 7.7 Hz, 1H), 7.03 (d, *J* = 7.5 Hz, 1H), 6.89 (t, *J* = 7.6 Hz, 1H), 6.62 (d, *J* = 10.4 Hz, 1H), 6.28 (dd, *J* = 10.4, 2.3 Hz, 1H), 5.73 – 5.51 (m, 2H), 5.29 (s, 1H), 5.11 (d, *J* = 10.4 Hz, 2H), 5.07 (d, *J* = 2.2 Hz, 1H), 4.81 (d, *J* = 17.2 Hz, 2H), 3.91 (d, *J* = 16.6 Hz, 1H), 3.72 (s, 4H), 2.70 (d, *J* = 16.7 Hz, 1H), 1.38 (s, 9H). **^{13}C NMR** (125 MHz, $CDCl_3$) δ 196.1, 157.6, 152.5, 142.1, 137.8, 136.9, 127.8, 127.5, 127.4, 127.0, 124.4, 120.7, 120.4, 119.2, 117.1, 98.1, 83.5, 52.2, 50.0, 36.6, 34.7, 29.8. **HRMS (ESI):** $[M+Na]^+$ calcd. for $C_{30}H_{33}NO_2^+$: 462.2404, found: 462.2409.

2-([1,1'-biphenyl]-4-yl)-8-(tert-butyl)-4'-(diallylamino)spiro[chromane-3,1'-cyclohexane]-2',4'-dien-6'-one (3r)

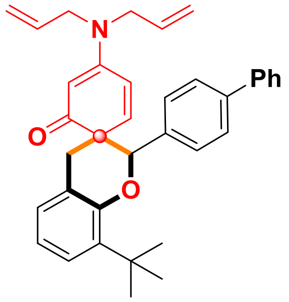

Flash column chromatography on a silica gel (ethyl acetate: petroleum ether, 1:3) afforded the product (43.3 mg, 84% yield) as a yellow oil.

^{1}H NMR (500 MHz, $CDCl_3$) δ 7.64 – 7.53 (m, 2H), 7.47 (d, J = 8.2 Hz, 2H), 7.50 – 7.39 (m, 4H), 7.37 – 7.31 (m, 1H), 7.20 (dd, J = 7.8, 1.7 Hz, 1H), 7.04 (dd, J = 7.6, 1.5 Hz, 1H), 6.89 (t, J = 7.6 Hz, 1H), 6.64 (d, J = 10.4 Hz, 1H), 6.30 (dd, J = 10.4, 2.3 Hz, 1H), 5.59 (s, 2H), 5.33 (s, 1H), 5.12 – 5.01 (m, 3H), 4.81– 4.77 (m, 2H), 3.93 (d, J = 16.4 Hz, 1H), 3.71 (s, 4H), 2.71 (d, J = 16.6 Hz, 1H), 1.39 (s, 9H). **^{13}C NMR** (125 MHz, $CDCl_3$) δ 196.1, 157.7, 152.6, 142.2, 140.9, 140.3, 137.9, 136.2, 128.8, 127.9, 127.9, 127.2, 126.9, 125.8, 124.5, 120.8, 120.5, 119.4, 98.3, 83.4, 77.3, 50.2, 36.7, 34.8, 29.9. **HRMS (ESI):** $[M+Na]^+$ calcd. for $C_{36}H_{37}NNaO_2^+$: 538.2717, found: 538.2715.

2-([1,1'-biphenyl]-4-yl)-8-(tert-butyl)-4'-(diethylamino)spiro[chromane-3,1'-cyclohexane]-2',4'-dien-6'-one (3s)

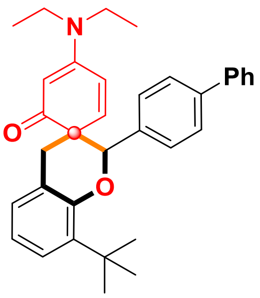

Flash column chromatography on a silica gel (ethyl acetate: petroleum ether, 1:3) afforded the product (40.7 mg, 83% yield) as a white oil.

^{1}H NMR (500 MHz, $CDCl_3$) δ 7.63 – 7.55 (m, 2H), 7.52 – 7.41 (m, 6H), 7.39 – 7.32 (m, 1H), 7.23 (dd, J = 7.7, 1.7 Hz, 1H), 7.07 (dd, J = 7.5, 1.6 Hz, 1H), 6.93 (t, J = 7.6 Hz, 1H), 6.69 (d, J = 10.4 Hz, 1H), 6.39 (dd, J = 10.5, 2.3 Hz, 1H), 5.39 (s, 1H), 5.07 (d, J = 2.3 Hz, 1H), 3.98 (dd, J = 16.5, 1.0 Hz, 1H), 3.19 (q, J = 7.0 Hz, 4H), 2.74 (d, J = 16.6 Hz, 1H), 1.43 (s, 9H), 1.01 (t, J = 7.0 Hz, 6H). **^{13}C NMR** (125 MHz, $CDCl_3$) δ 195.3, 156.3, 152.6, 142.2, 141.0, 140.3, 137.9, 136.3, 128.8, 128.0, 127.9, 127.2, 126.9, 125.8, 124.5, 120.9, 120.5, 119.1, 97.5, 83.3, 49.9, 45.0, 36.8, 34.8, 29.9. **HRMS (ESI):** $[M+Na]^+$ calcd. for $C_{34}H_{37}NNaO_2^+$: 514.2717, found: 514.2719.

4'-(dibenzylamino)-8-isopropyl-2-phenylspiro[chromane-3,1'-cyclohexane]-2',4'-dien-6'-one (3t)

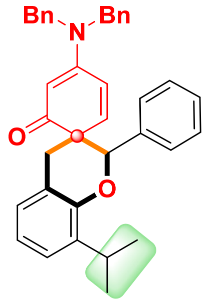

Flash column chromatography on a silica gel (ethyl acetate: petroleum ether, 1:5) afforded the product (25.7 mg, 49% yield) as a yellow oil.

^{1}H NMR (500 MHz, $CDCl_3$) δ 7.41 – 7.35 (m, 3H), 7.34 – 7.25 (m, 8H), 7.13 – 7.08 (m, 1H), 6.99 (dd, J = 8.0, 1.3 Hz, 1H), 6.90 (t, J = 7.6 Hz, 1H), 6.88 – 6.83 (m, 4H), 6.56 (d, J = 10.5 Hz, 1H), 6.36 (dd, J = 10.5, 2.3 Hz, 1H), 5.31 (s, 1H), 5.23 (d, J = 2.3 Hz, 1H), 4.36 (d, J = 13.0 Hz, 4H), 3.90 (dd, J = 16.5, 1.2 Hz, 1H), 3.38 – 3.30 (m, 1H), 2.71 (d, J = 16.6 Hz, 1H), 1.22 (dd, J = 14.5, 6.9 Hz, 6H). **^{13}C NMR** (125 MHz, $CDCl_3$) δ 196.5, 158.3, 150.8, 142.7, 137.3, 136.4, 128.9, 127.7, 127.6, 127.5, 127.3, 127.2, 126.3, 124.0, 120.7, 119.8, 119.2, 98.7, 83.5, 77.3, 50.3, 36.3, 26.9, 23.1, 22.3. **HRMS (ESI):** $[M+Na]^+$ calcd. for $C_{37}H_{35}NNaO_2^+$: 548.2560, found: 548.2568.

8-(sec-butyl)-4'-(dibenzylamino)-2-phenylspiro[chromane-3,1'-cyclohexane]-2',4'-dien-6'-one (3u)

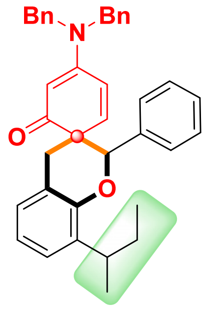

Flash column chromatography on a silica gel (ethyl acetate: petroleum ether, 1:5) afforded the product (36.1 mg, 67% yield, dr 2:1) as a yellow oil.

^{1}H NMR (500 MHz, $CDCl_3$) δ 7.41 – 7.34 (m, 3H), 7.35 – 7.20 (m, 8H), 7.09 – 7.04 (m, 1H), 7.02 – 6.94 (m, 1H), 6.98 – 6.89 (m, 1H), 6.87 – 6.80 (m, 4H), 6.55 (m, 1H), 6.39 – 6.30 (m, 1H), 5.31 (d, J = 3.1 Hz, 1H), 5.23 (t, J = 2.8 Hz, 1H), 4.58 – 4.23 (m, 4H), 3.96 – 3.84 (m, 1H), 3.16 – 3.09 (m, 1H), 2.71 (d, J = 16.6 Hz, 1H), 1.66 – 1.48 (m, 2H), 1.20 (m, 3H), 0.86 (t, J = 7.4 Hz, 2H), 0.79 (t, J = 7.4 Hz, 1H). **^{13}C NMR** (125 MHz, $CDCl_3$) δ 196.6, 196.6, 158.4, 158.4, 151.2, 151.2, 142.9, 142.8, 137.3, 135.2, 135.2, 128.9, 128.6, 127.7, 127.6, 127.6, 127.5, 127.5, 127.3, 127.3, 127.1, 126.3, 124.8, 120.7, 119.8, 119.7, 119.2, 119.1, 98.7, 98.7, 83.6, 83.4, 77.3, 50.3, 36.3, 33.9, 33.3, 30.7, 29.5, 20.9, 20.3, 12.4, 12.2. **HRMS (ESI):** $[M+Na]^+$ calcd. for $C_{38}H_{37}NNaO_2^+$: 562.2717, found: 562.2723.

8-(tert-butyl)-4'-(diethylamino)-2-ethylspiro[chromane-3,1'-cyclohexane]-2',4'-dien-6'-one (4k)

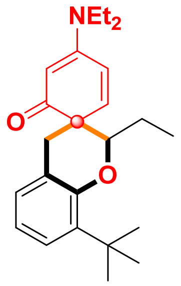

Flash column chromatography on a silica gel (ethyl acetate: petroleum ether, 1:3) afforded the product (30.1 mg, 82% yield, dr 1:1) as a white solid.

^{1}H NMR (400 MHz, $CDCl_3$) δ 7.13 (m, 2H), 6.93 (m, 2H), 6.79 (m, 2H), 6.02 (m, 1H), 5.64 (m, 1H), 5.30 (m, 1H), 5.21 (m, 2H), 5.04 (m, 1H), 4.37 (m, 1H), 3.44 – 3.21 (m, 7H), 3.19 – 3.02 (m, 2H), 2.75 (m, 2H), 2.42 (m, 4H), 2.01 – 1.93 (m, 2H), 1.78 – 1.59 (m, 4H), 1.41 (m, 16H), 1.18 (m, 13H). **^{13}C NMR** (100 MHz, $CDCl_3$) δ 198.7, 163.1, 162.9, 137.1, 134.4, 128.1, 127.9, 124.3, 121.5, 121.3, 119.9, 119.3, 116.4, 98.5, 98.3, 82.3, 79.4, 44.2, 42.3, 42.0, 34.8, 34.7, 34.5, 34.0, 29.7, 29.6, 23.5, 22.8, 22.6, 22.6, 12.2. **HRMS (ESI):** $[M+Na]^+$ calcd. for $C_{24}H_{33}NNaO_2^+$: 390.2404, found: 390.2411.

6,6'-((3-(tert-butyl)-2-((3,5-dimethoxybenzyl)oxy)phenyl)methylene)bis(3-(diethylamino)phenol) (5a)

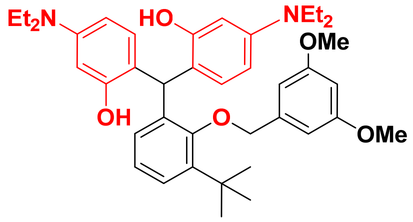

Flash column chromatography on a silica gel (ethyl acetate: petroleum ether, 1:15) afforded the product (51.8 mg, 81% yield) as a white solid.

^{1}H NMR (500 MHz, $CDCl_3$) δ 7.29 (m, 1H), 7.14 (dd, J = 7.7, 1.8 Hz, 1H), 7.09 (t, J = 7.7 Hz, 1H), 6.94 (d, J = 8.3 Hz, 2H), 6.56 (d, J = 2.4 Hz, 2H), 6.44 (t, J = 2.3 Hz, 1H), 6.22 (d, J = 7.9 Hz, 4H), 5.76 (s, 1H), 5.19 (s, 2H), 4.75 (s, 2H), 3.81 (s, 6H), 3.31 (q, J = 7.1 Hz, 8H), 1.50 (s, 9H), 1.15 (t, J = 7.0 Hz, 12H). **^{13}C NMR** (100 MHz, $CDCl_3$) δ 160.9, 155.5, 148.6, 139.3, 135.4, 130.7, 129.4, 125.7, 124.4, 114.3, 105.2, 104.6, 100.3, 100.0, 76.6, 55.3, 44.3, 37.4, 35.4, 31.3, 12.7. **HRMS (ESI):** $[M+Na]^+$ calcd. for $C_{40}H_{52}N_2NaO_5^+$: 663.3768, found: 663.3778.

Reference:

1. Jurberg, I. D.; Peng, B.; Wçstefeld, E.; Wasserloos, M.; Maulide, N. Intramolecular Redox-Triggered C-H Functionalization. *Angew. Chem. Int. Ed.*, **2012**, *51*, 1950-1953.

5. In Vitro Antifungal Activities

Each target compound was dissolved in acetone to prepare the stock solution (2.5 g/L). The stock solution was added into the PDA medium, and the concentration of target compound in the medium was 200.0 mg/L. Pure acetone without the target compound was utilized as the blank control, and difenoconazole and thifluzamide were coassayed as the reference compound. Fresh dishes with a diameter of 6 mm were taken from the edge of the PDA cultured fungi colonies and inoculated on the above three PDA media. Each treatment was tested for three replicates, and the antifungal effect was averaged. The relative inhibitory rate

I (%) of all the tested compounds was calculated through the equation: $I\ (\%) = [(C-T)/(C-5)]\ \mathrm{x}100$. In this equation, I is the inhibitory rate and C and T are the colony diameter of the blank control (mm) and treatment (mm), respectively. Mycelia growth of four crop pathogenic fungi after treating with the target compounds on PDA medium as illustrated in the figures (Figure S1.) below.

Table S1. In Vitro Antifungal Activities of Spiro[chromane-3,1'-cyclohexane]-2',4'-dien-6'-ones at 200.0 mg/L

Compound	Inhibition rate (%)[a]			
	Rhizoctonia solani	*Alternaria solani*	*Alternaria mali*	*Sclerotium rolfsii*
**3u**	9.28	8.09	7.08	0.00
**3a**	40.21	60.35	53.56	29.09
**3k**	19.59	0.00	0.00	18.18
**3t**	0.00	12.50	0.00	0.91
**3q**	19.59	61.35	55.98	8.18
**3j**	25.77	45.59	30.97	20.91
**Thifluzamide**	87.63	-	-	96.36
**Difenoconazole**	-	94.85	100.00	-

[a]Values are based on three replicates.

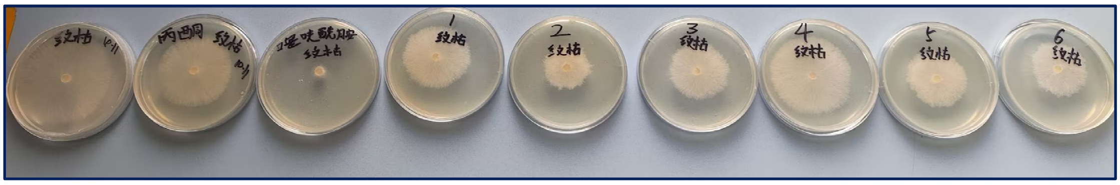

(A). *Rhizoctonia solani*

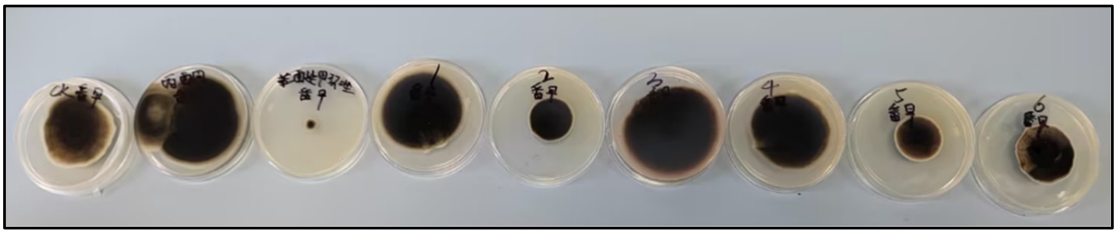

(B). *Alternaria solani*

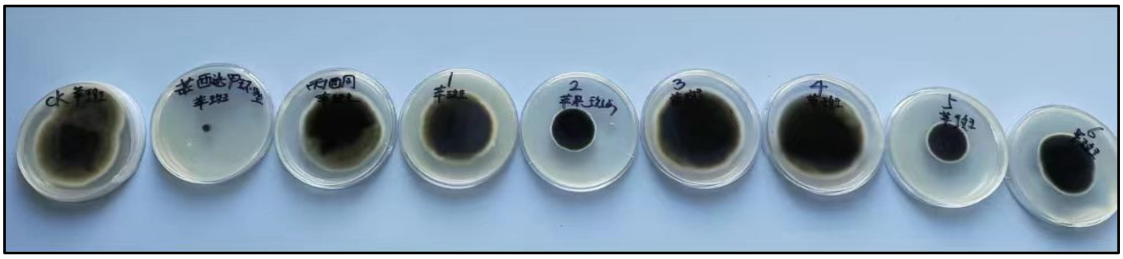

(C). *Alternaria mali*

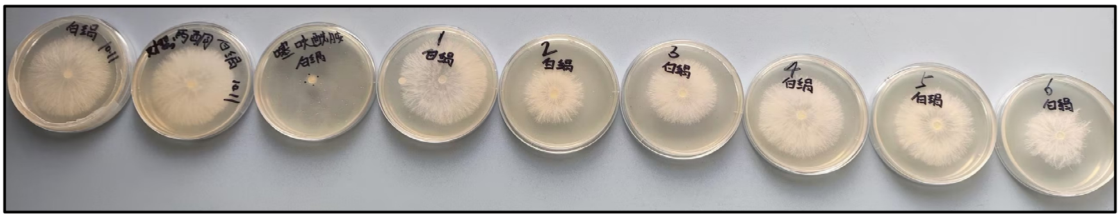

(D). *Sclerotium rolfsii.*

Figure S1. (A). *Rhizoctonia solani;* **(B).** *Alternaria solani;* **(C).** *Alternaria mali;* **(D).** *Sclerotium rolfsii.*

6. Crystal Structures and Data

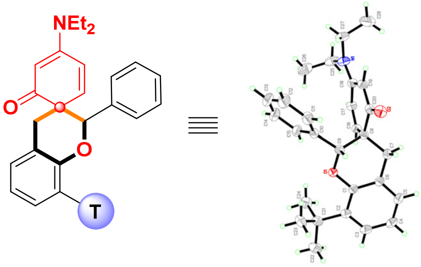

3a **(CCDC 2310751)**

Table S2. Crystal data and structure refinement for **3a**.

Identification code	**3a**
Empirical formula	$C_{28}H_{33}NO_2$
Formula weight	415.55
Temperature	293(2) K
Wavelength	1.54184 A
Crystal system, space group	Orthorhombic, Pbca
Unit cell dimensions	a = 16.5265(4) A alpha = 90 deg.
	b = 11.9941(3) A beta = 90 deg.
	c = 24.1078(5) A gamma = 90 deg.
Volume	4778.66(19) A^3
Z, Calculated density	8, 1.155 Mg/m^3
Absorption coefficient	0.556 mm^-1
F(000)	1792
Crystal size	0.230 x 0.220 x 0.210 mm
Theta range for data collection	3.667 to 67.237 deg.
Limiting indices	-19<=h<=19, -14<=k<=10, -28<=l<=15
Reflections collected / unique	11017 / 4284 [R(int) = 0.0250]

Completeness to theta = 67.237	99.9 %
Refinement method	Full-matrix least-squares on F^2
Data / restraints / parameters	4284 / 0 / 286
Goodness-of-fit on F^2	1.033
Final R indices [I>2sigma(I)]	R1 = 0.0418, wR2 = 0.1044
R indices (all data)	R1 = 0.0546, wR2 = 0.1132
Extinction coefficient	0.00151(11)
Largest diff. peak and hole	0.206 and -0.148 e.A^-3

7. ^{1}H and ^{13}C NMR Spectra

8-(tert-butyl)-4'-(diethylamino)-2-phenylspiro[chromane-3,1'-cyclohexane]-2',4'-dien-6'-one (3a)

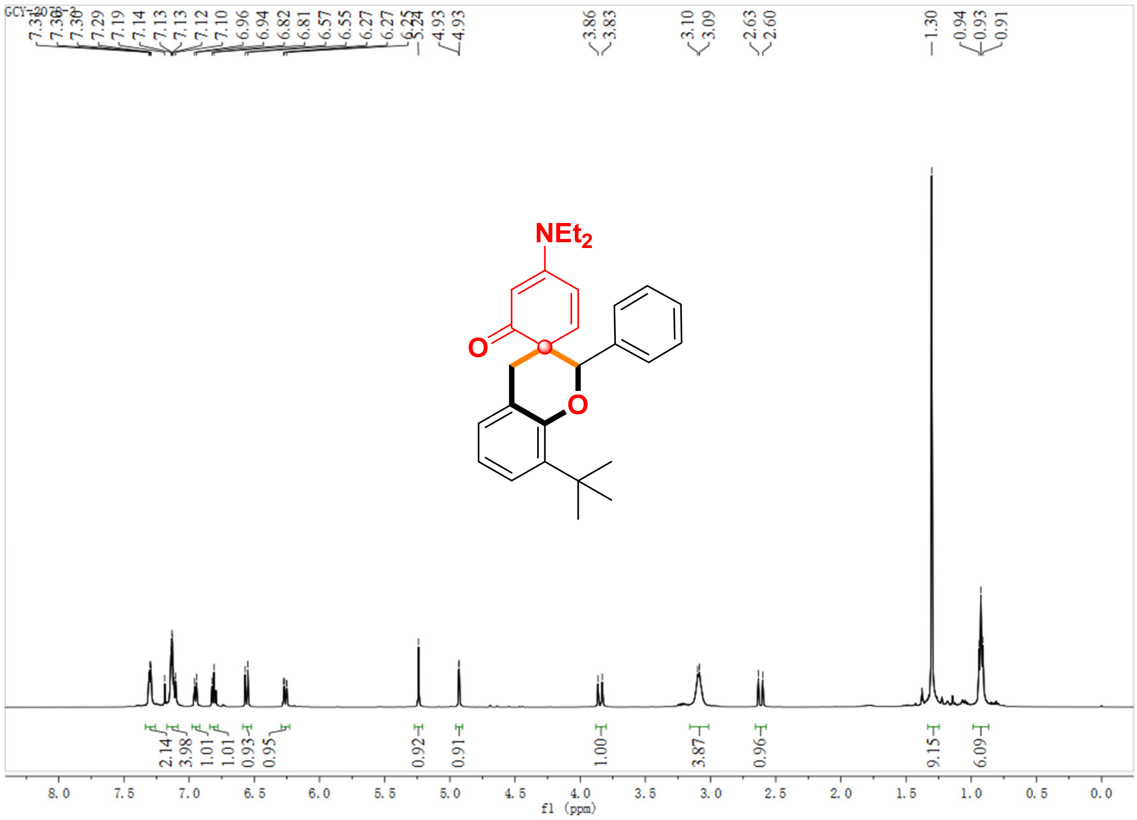

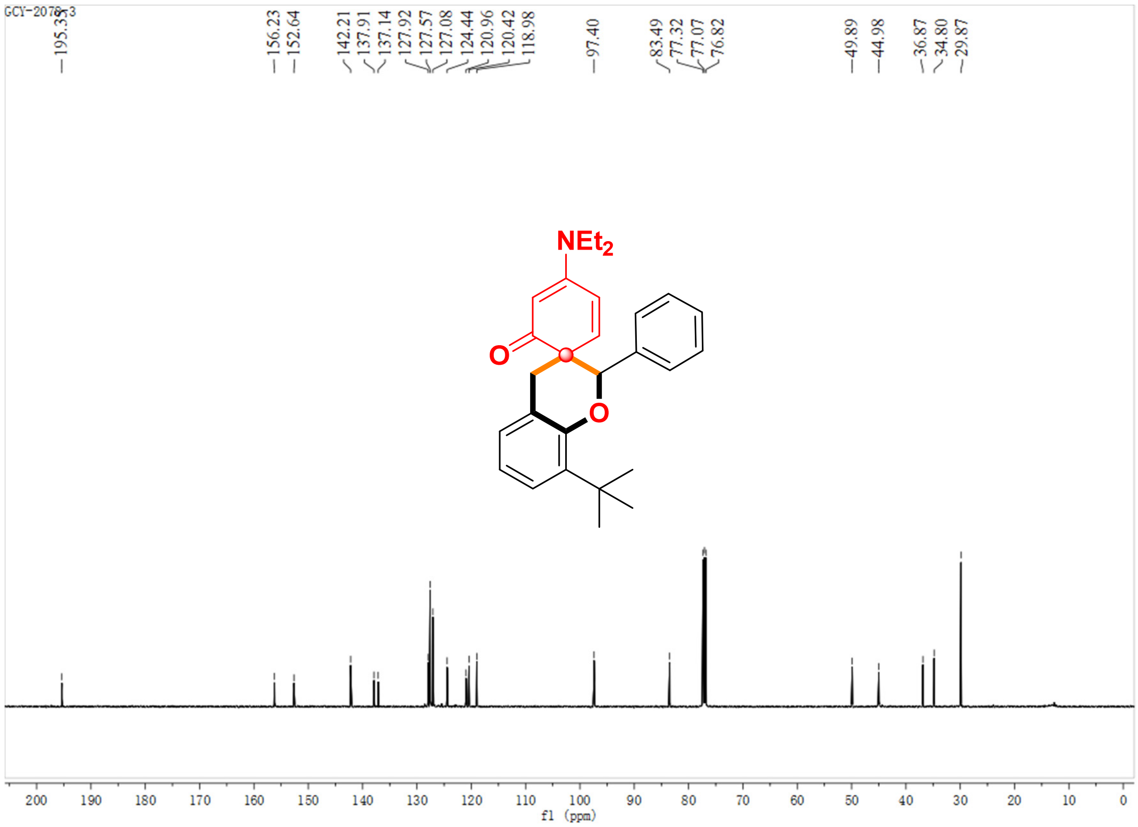

8-(tert-butyl)-4'-(diethylamino)-2-(2-fluorophenyl)spiro[chromane-3,1'-cyclohexane]-2',4'-dien-6'-one (3b)

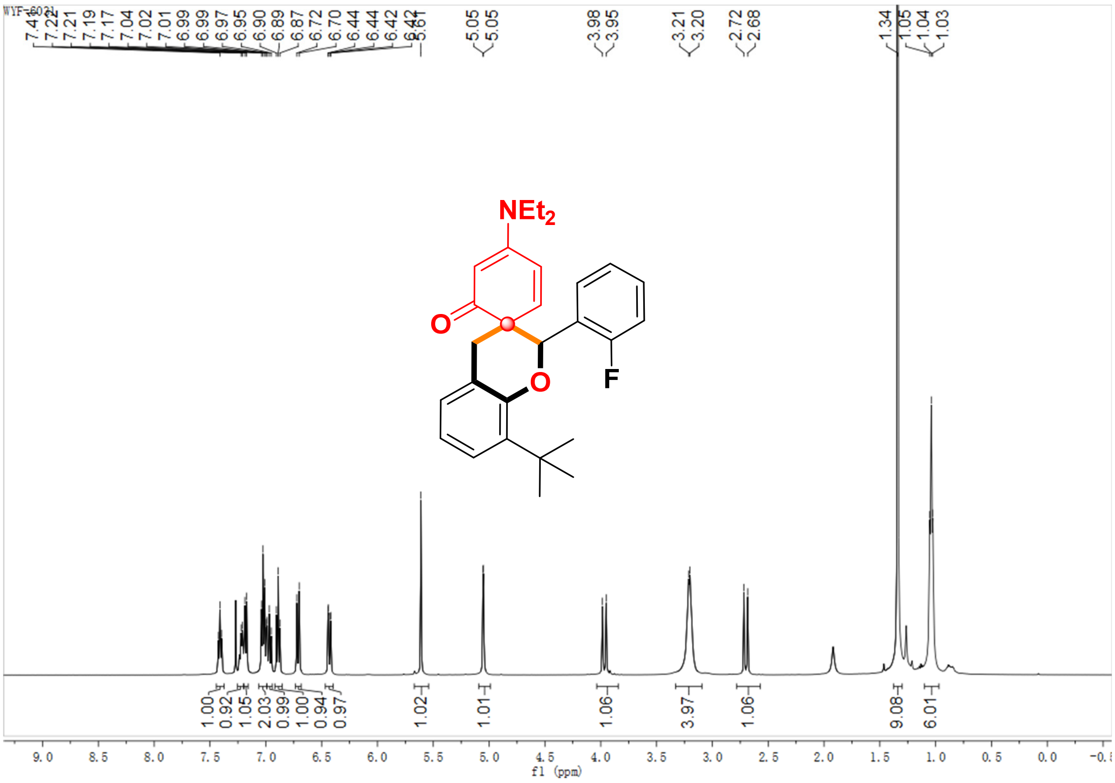

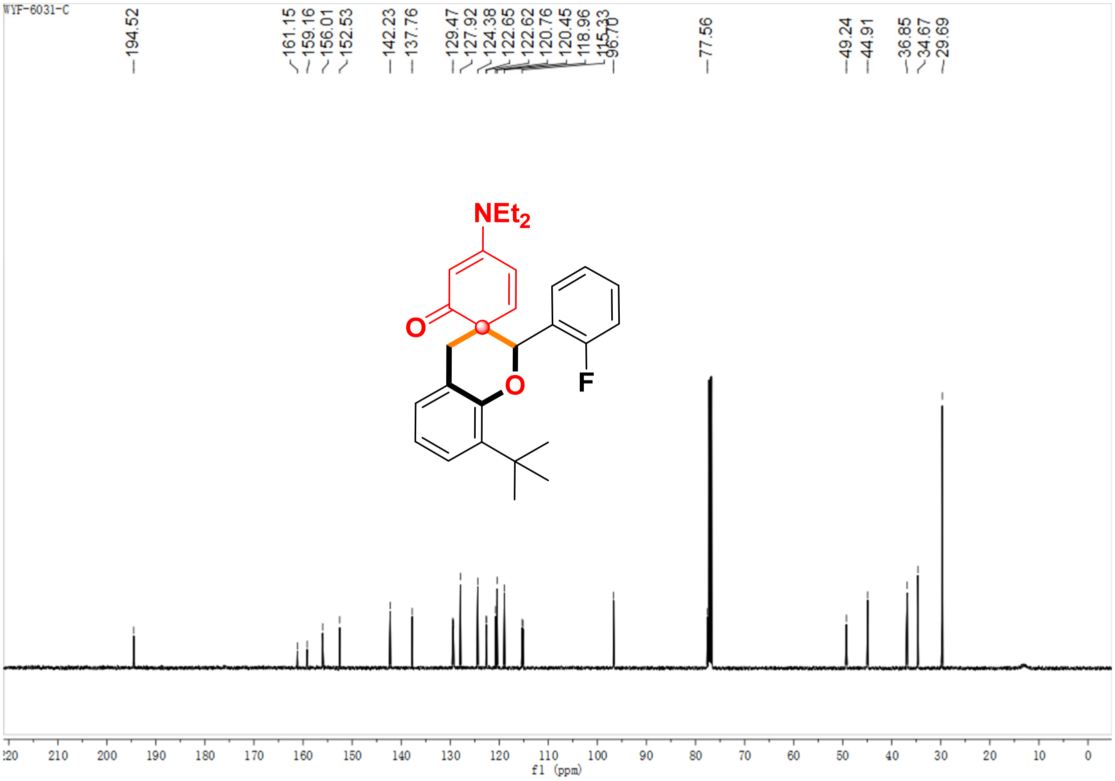

8-(tert-butyl)-2-(3-chlorophenyl)-4'-(diethylamino)spiro[chromane-3,1'-cyclohexane]-2',4'-dien-6'-one (3c)

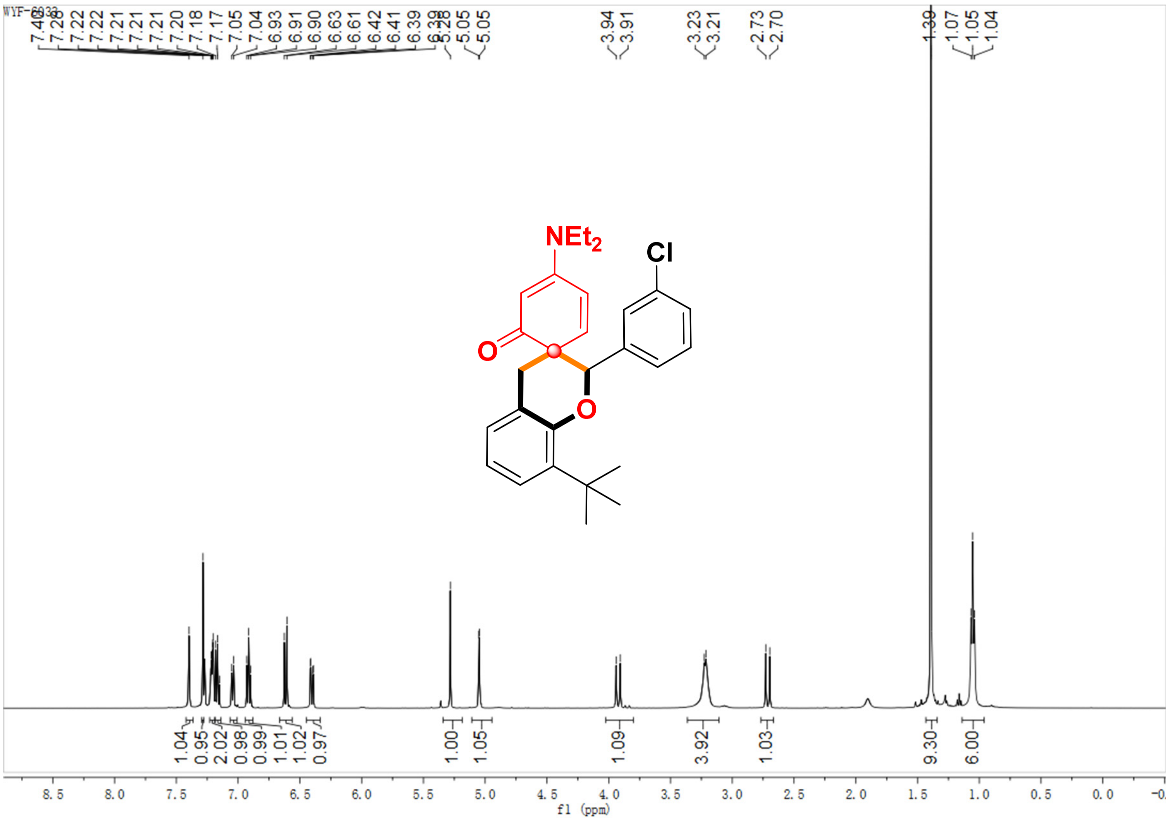

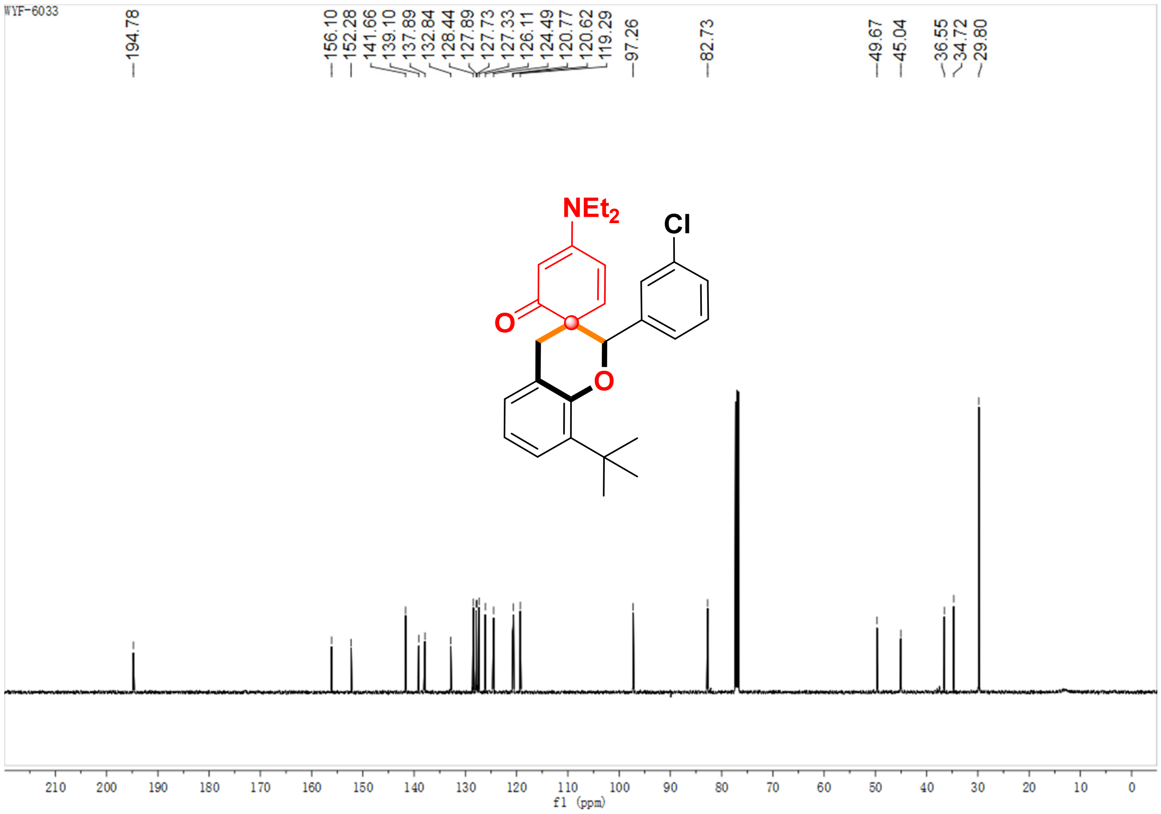

8-(tert-butyl)-4'-(diethylamino)-2-(m-tolyl)spiro[chromane-3,1'-cyclohexane]-2',4'-dien-6'-one (3d)

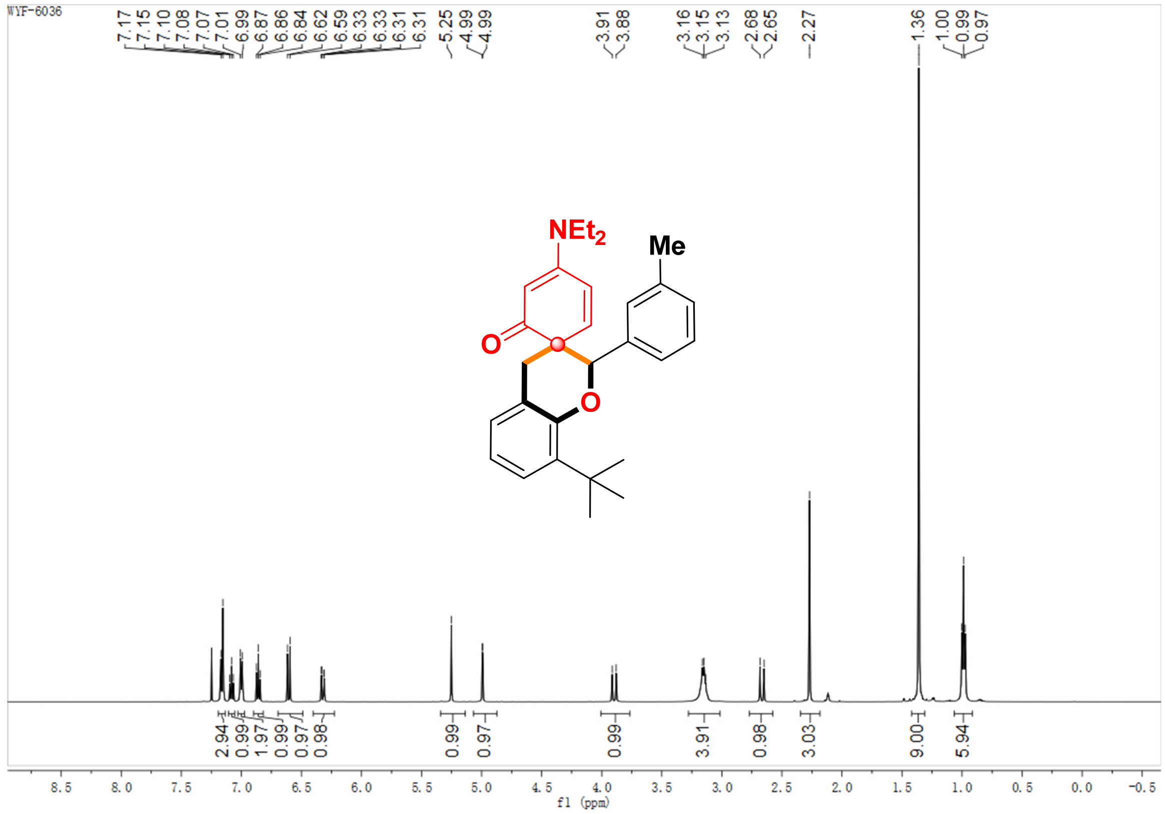

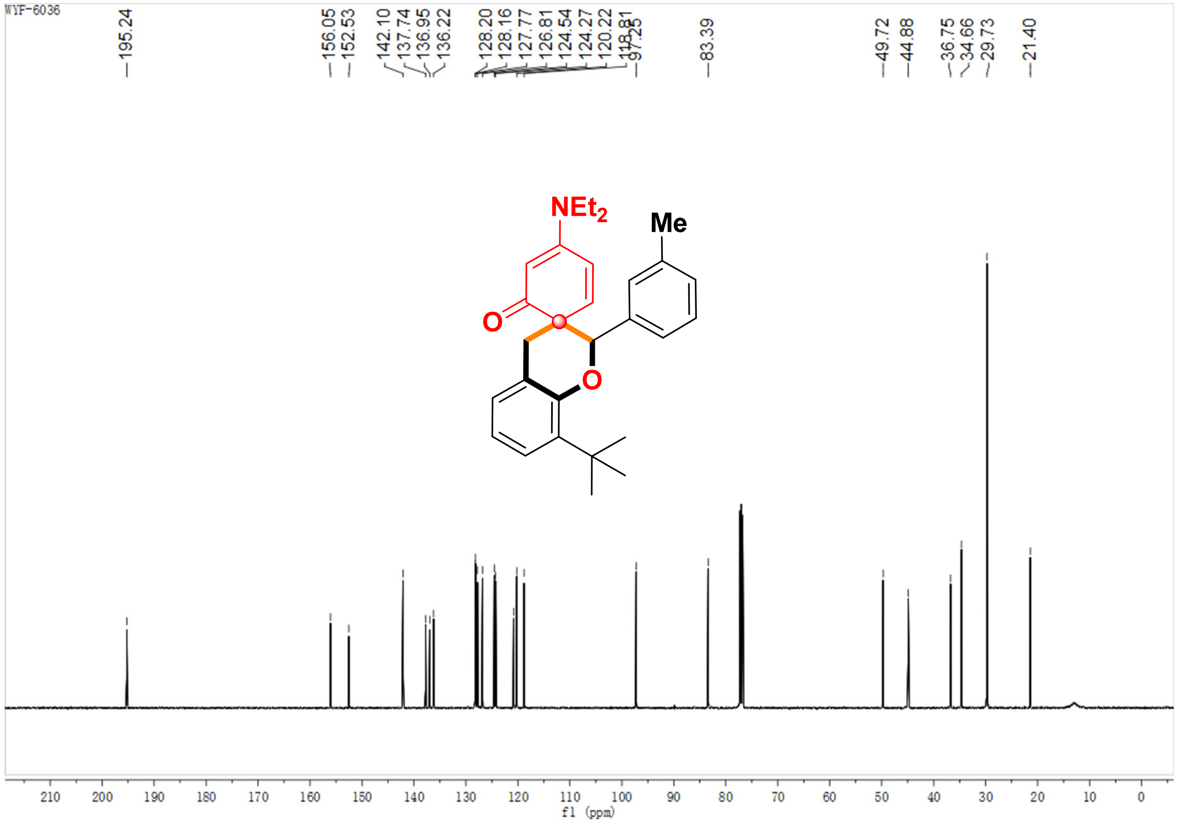

8-(tert-butyl)-4'-(diethylamino)-2-(p-tolyl)spiro[chromane-3,1'-cyclohexane]-2',4'-dien-6'-one (3e)

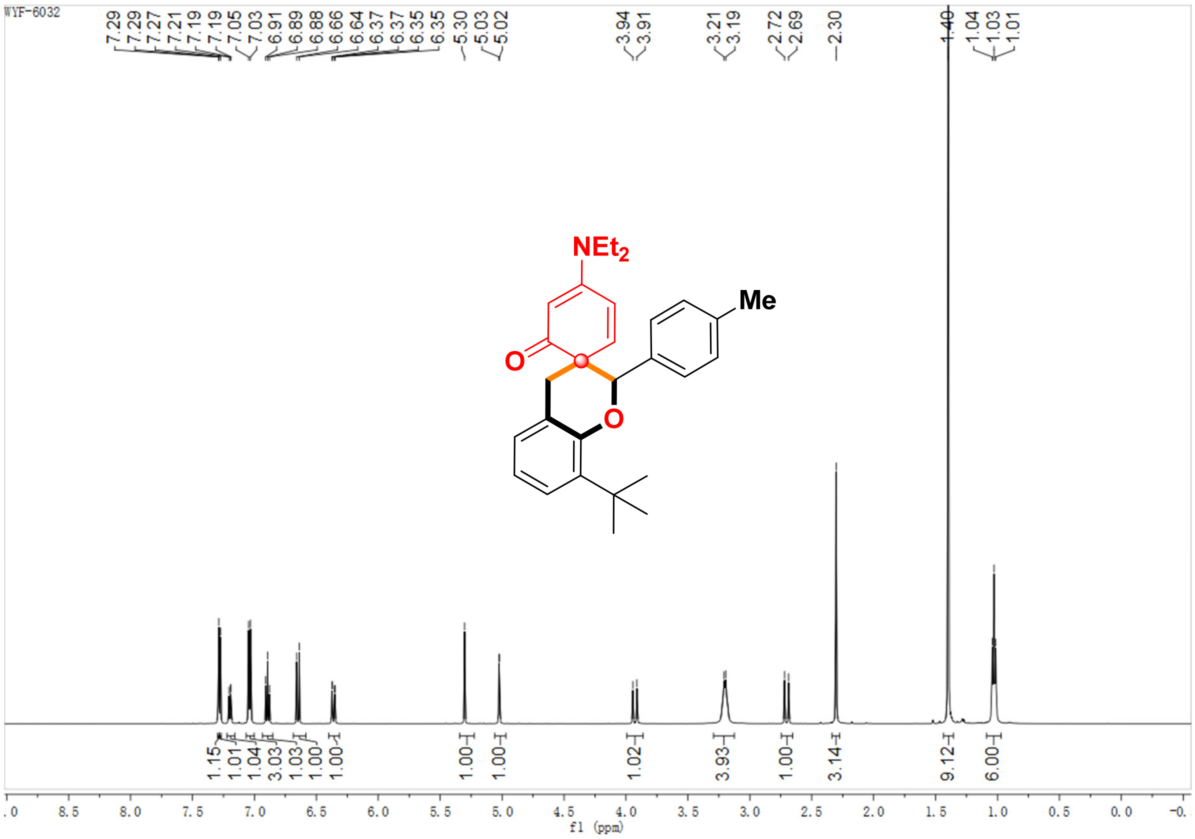

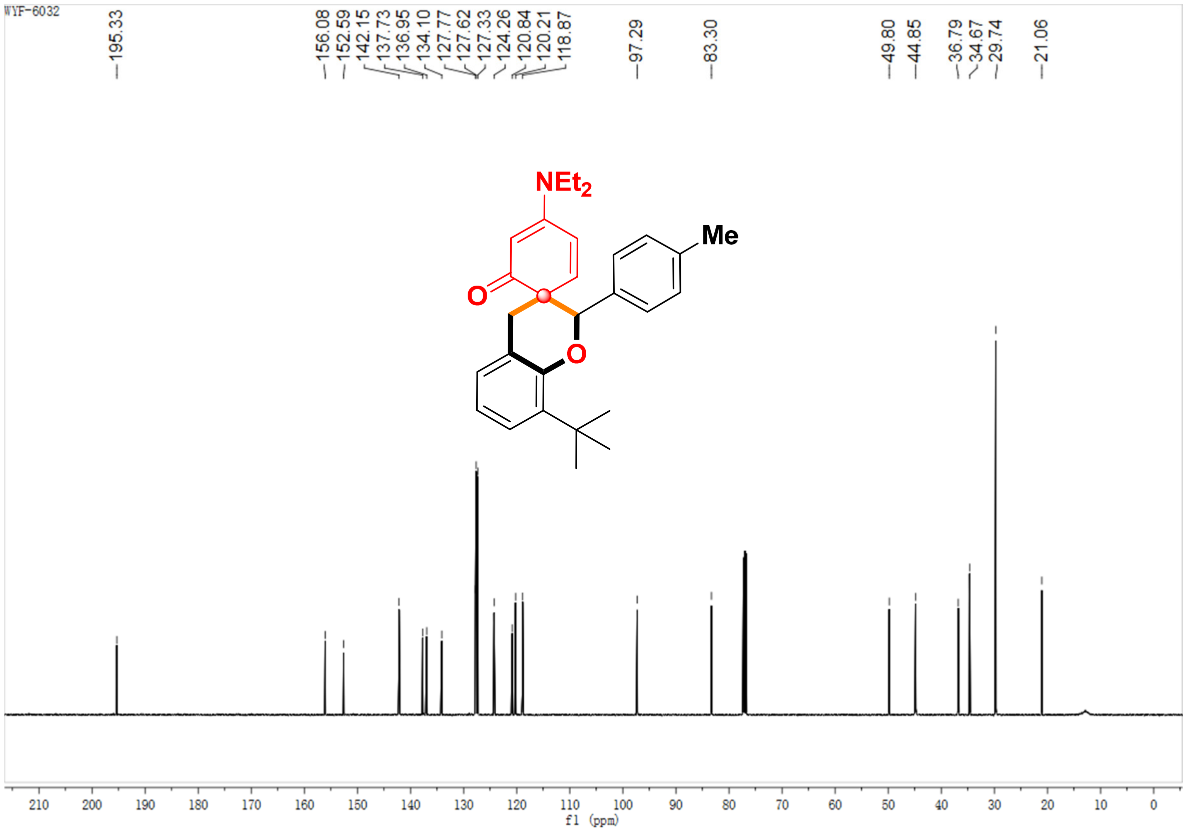

8-(tert-butyl)-4'-(diethylamino)-2-(4-fluorophenyl)spiro[chromane-3,1'-cyclohexane]-2',4'-dien-6'-one (3f)

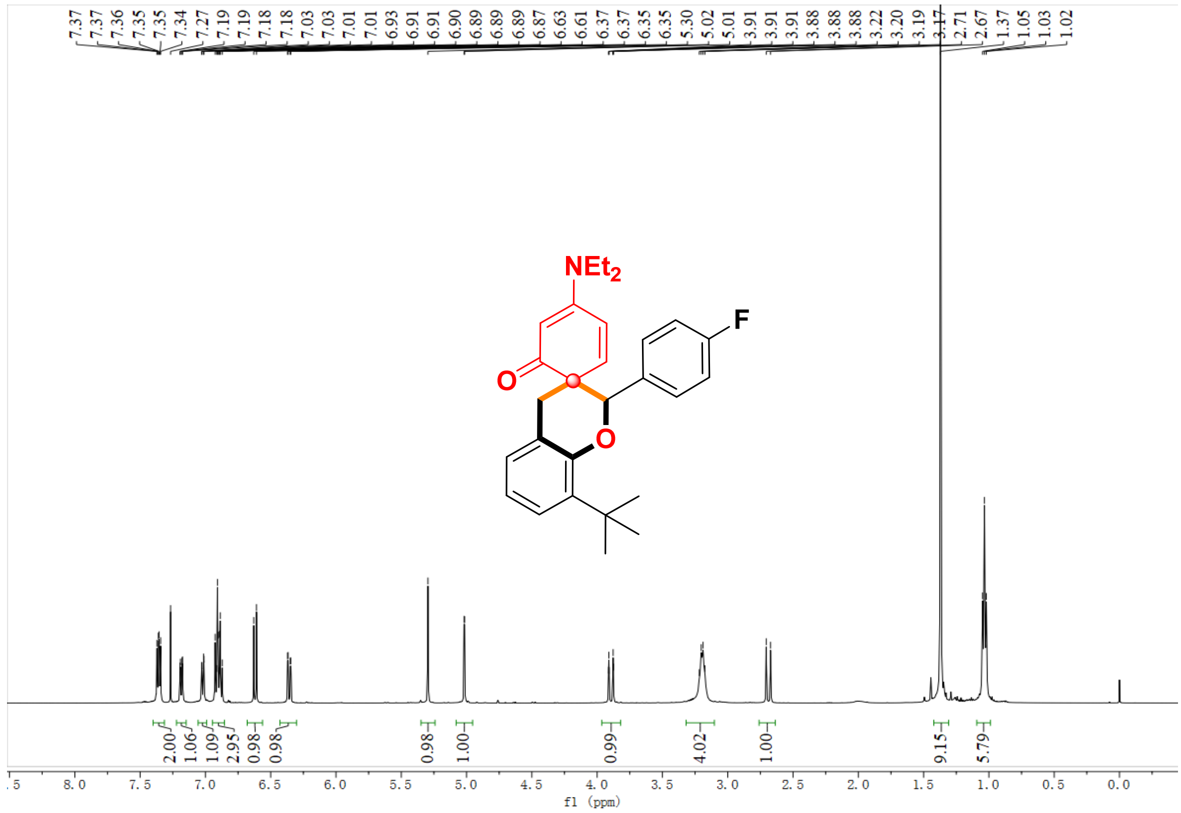

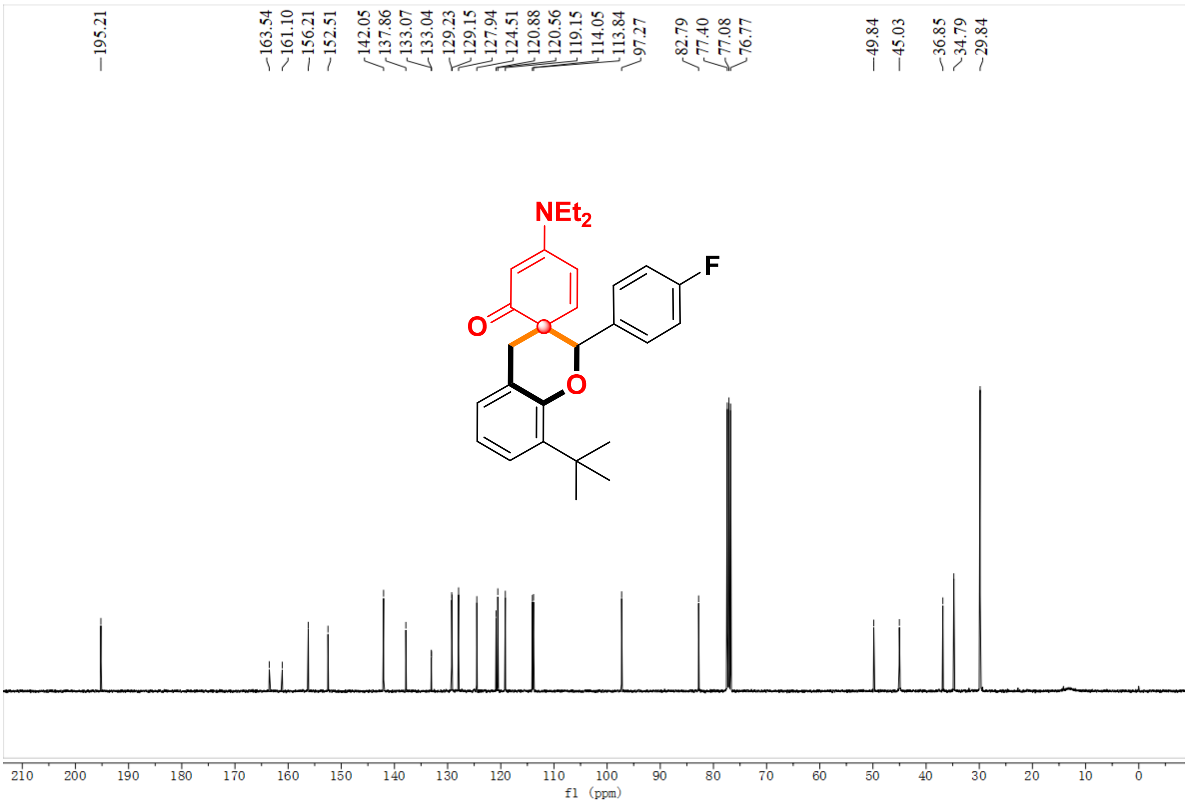

2-(4-bromophenyl)-8-(tert-butyl)-4'-(diethylamino)spiro[chromane-3,1'-cyclohexane]-2',4'-dien-6'-one (3g)

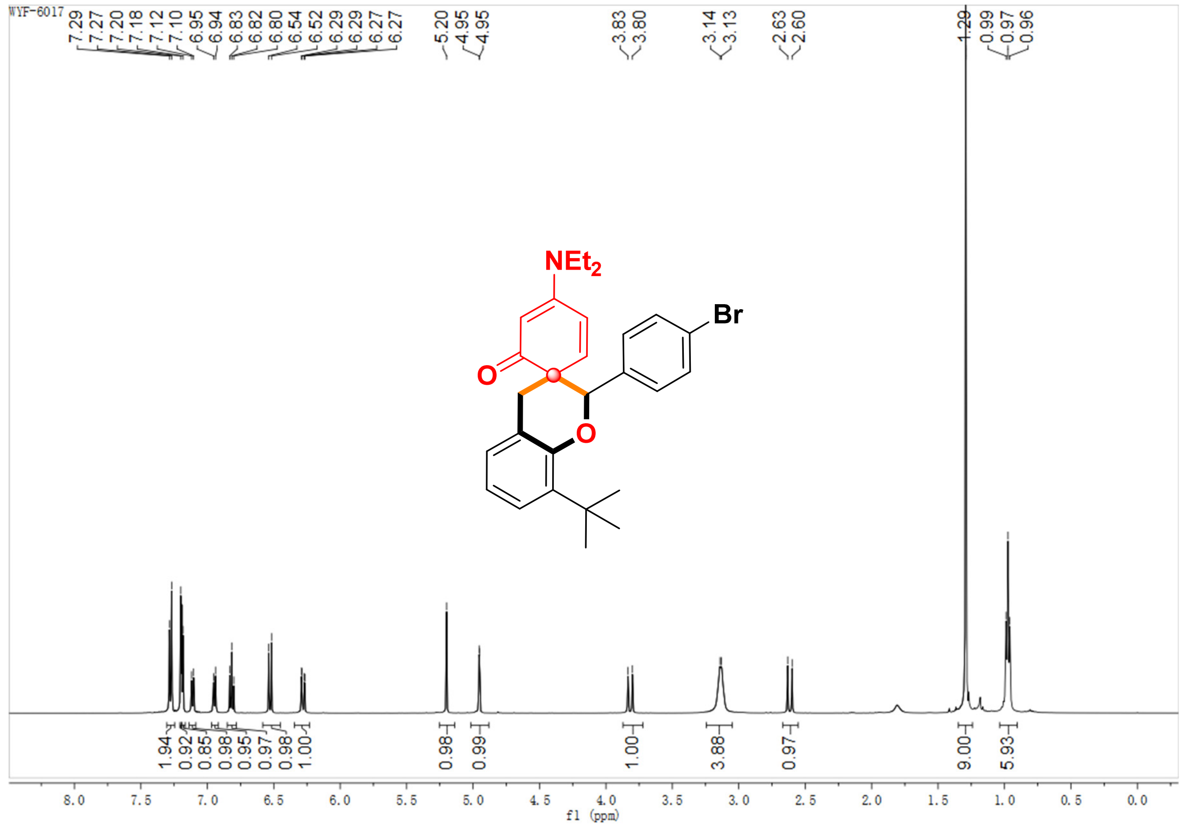

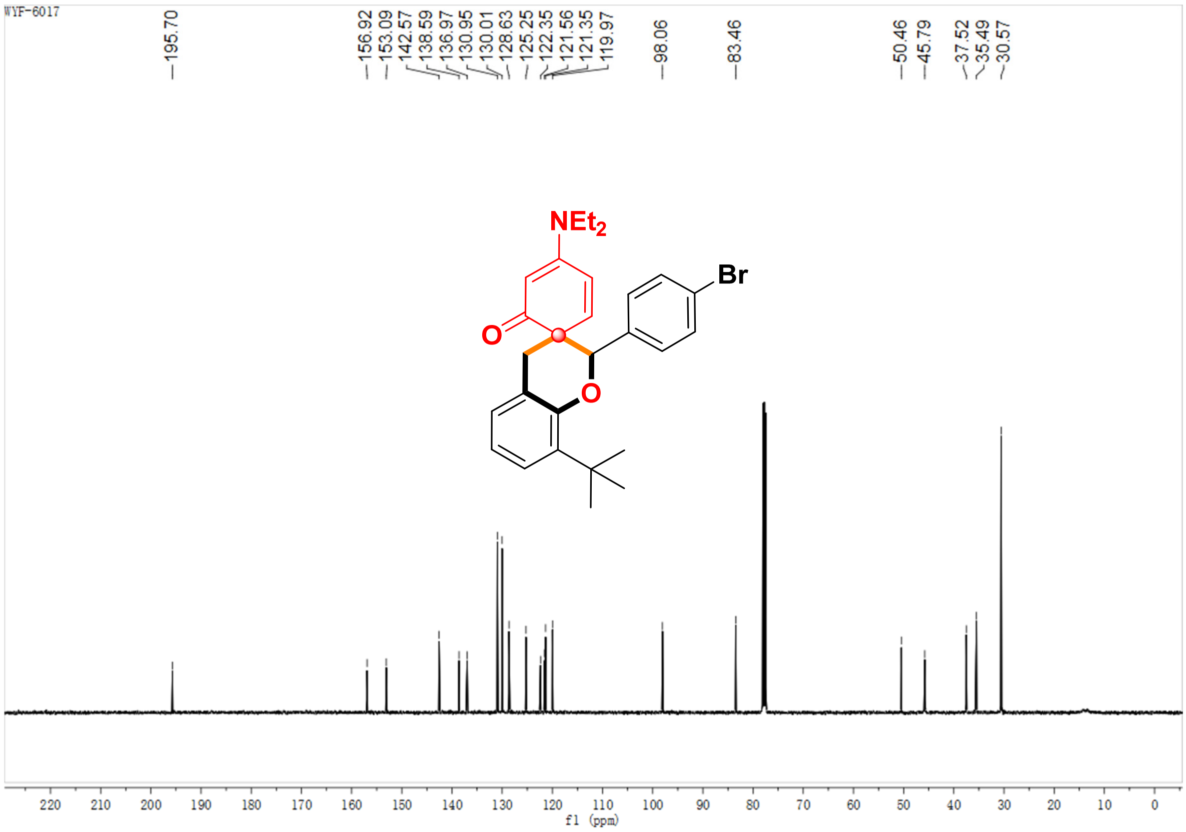

8-(tert-butyl)-4'-(diethylamino)-2-(naphthalen-2-yl)spiro[chromane-3,1'-cyclohexane]-2',4'-dien-6'-one (3h)

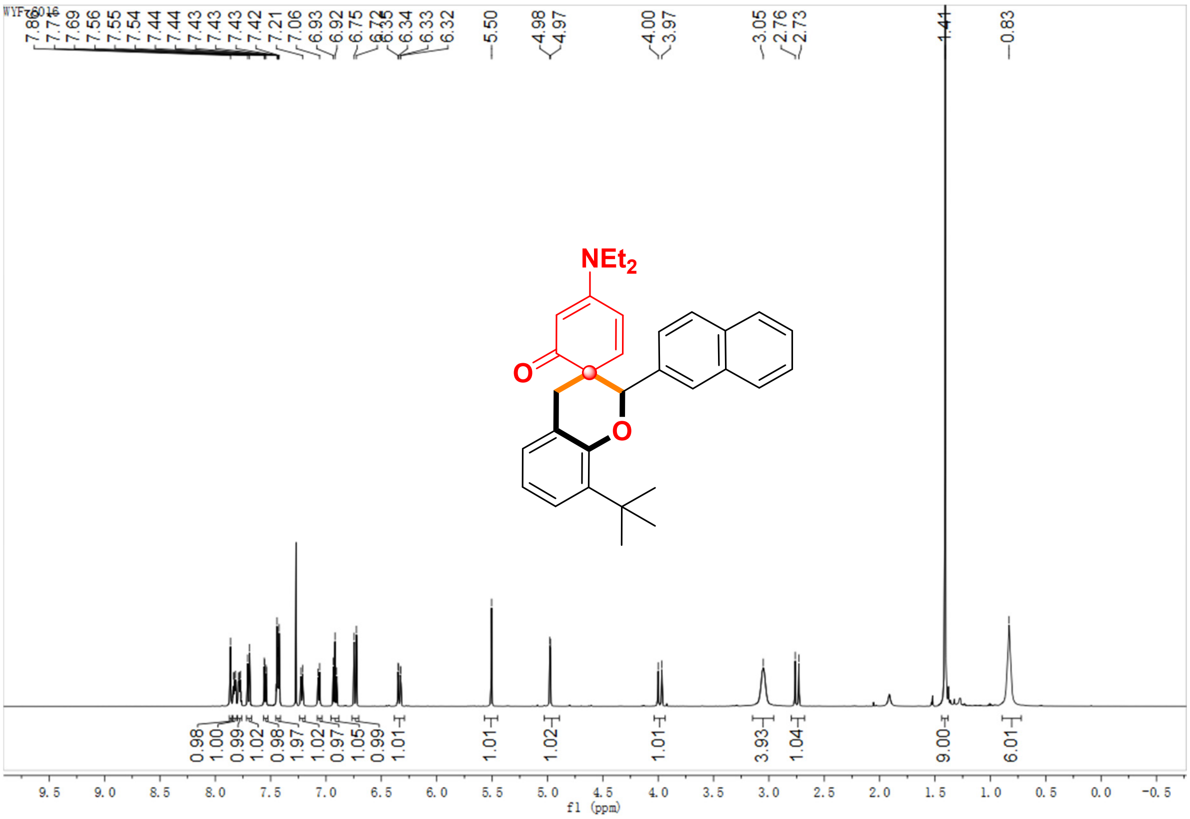

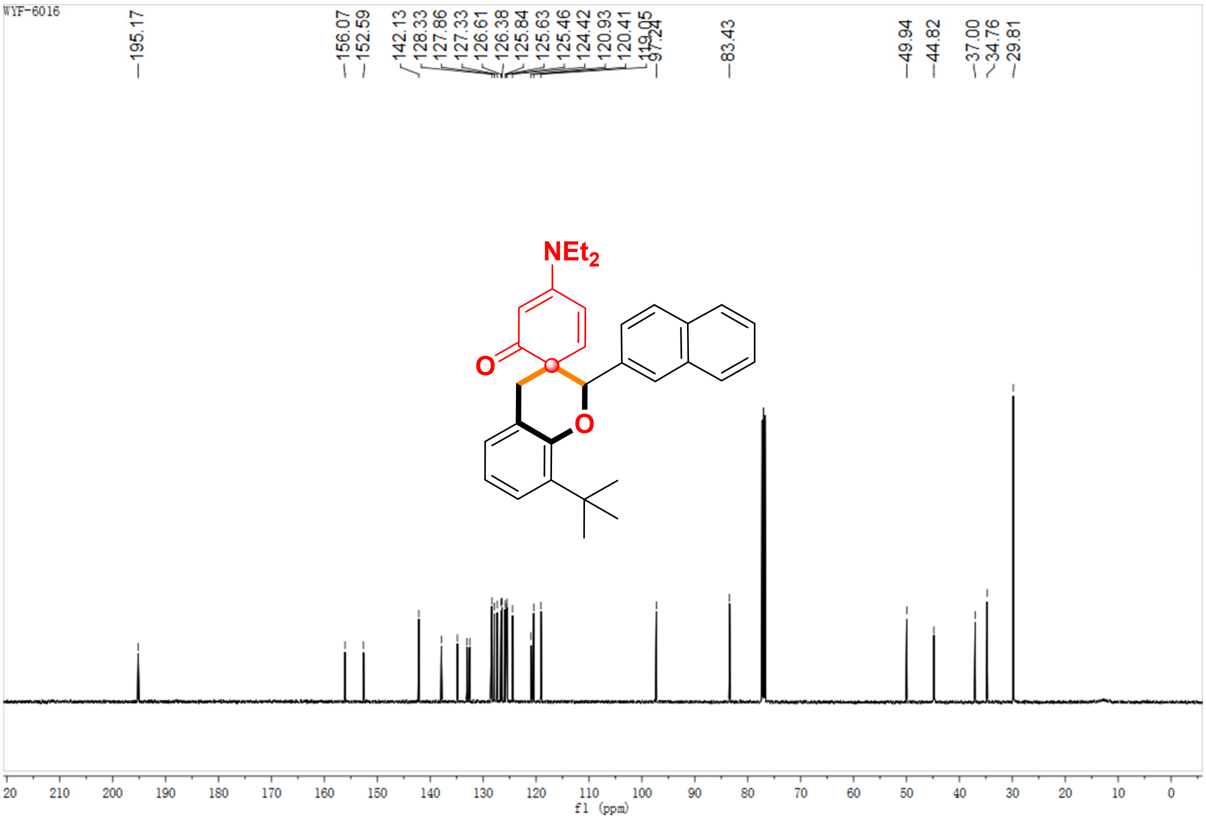

8-(tert-butyl)-4'-(diethylamino)-2,2-dimethylspiro[chromane-3,1'-cyclohexane]-2',4'-dien-6'-one (3i)

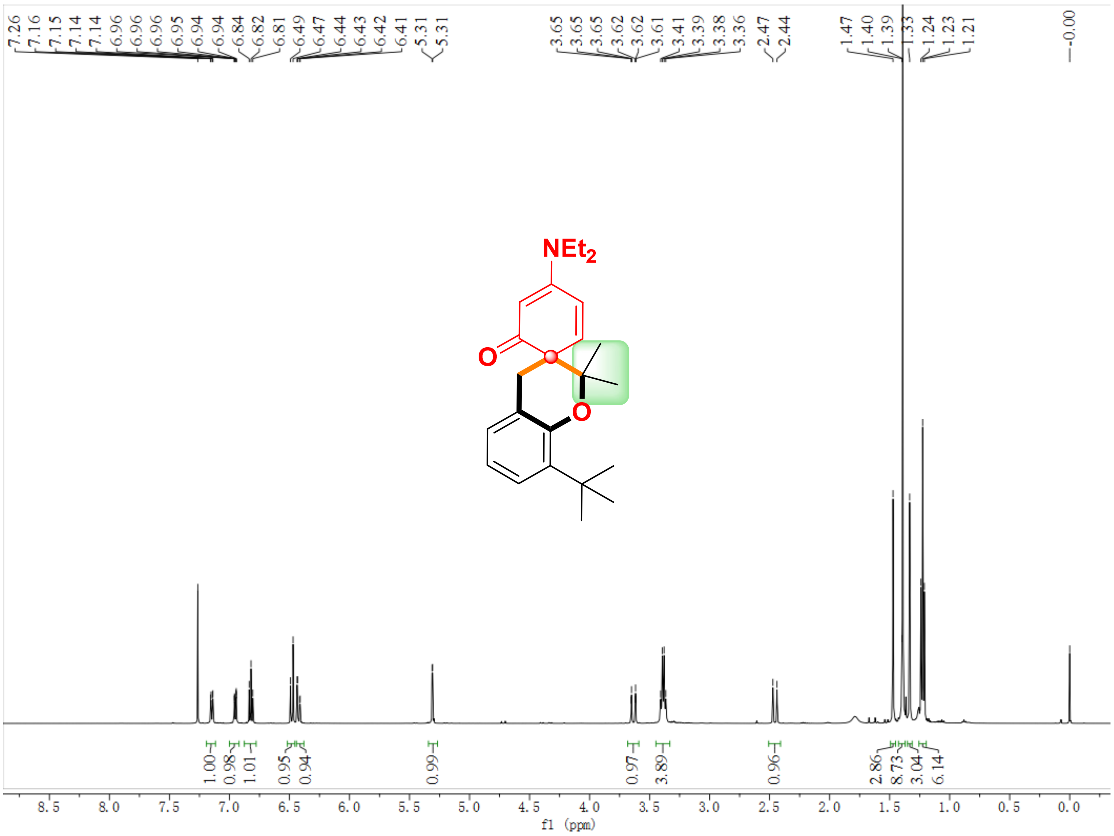

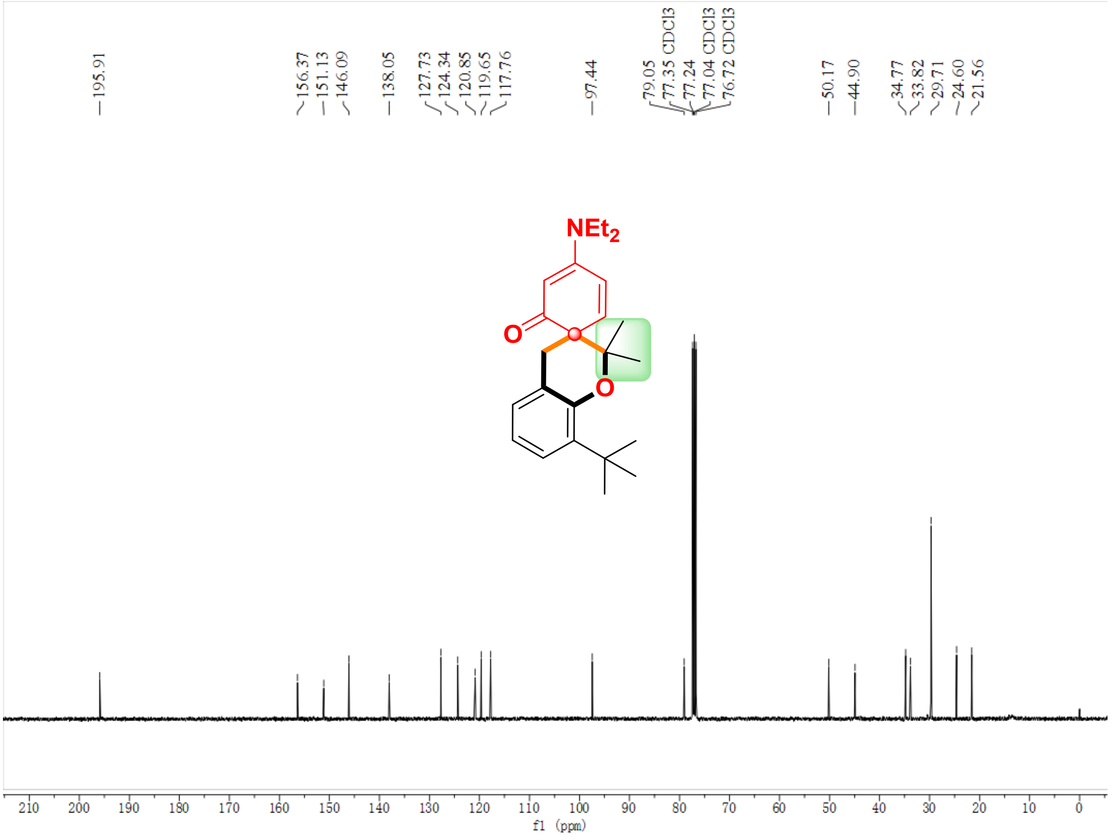

8-(tert-butyl)-4'-(diethylamino)-2-methyl-2-phenylspiro[chromane-3,1'-cyclohexane]-2',4'-dien-6'-one (3j)

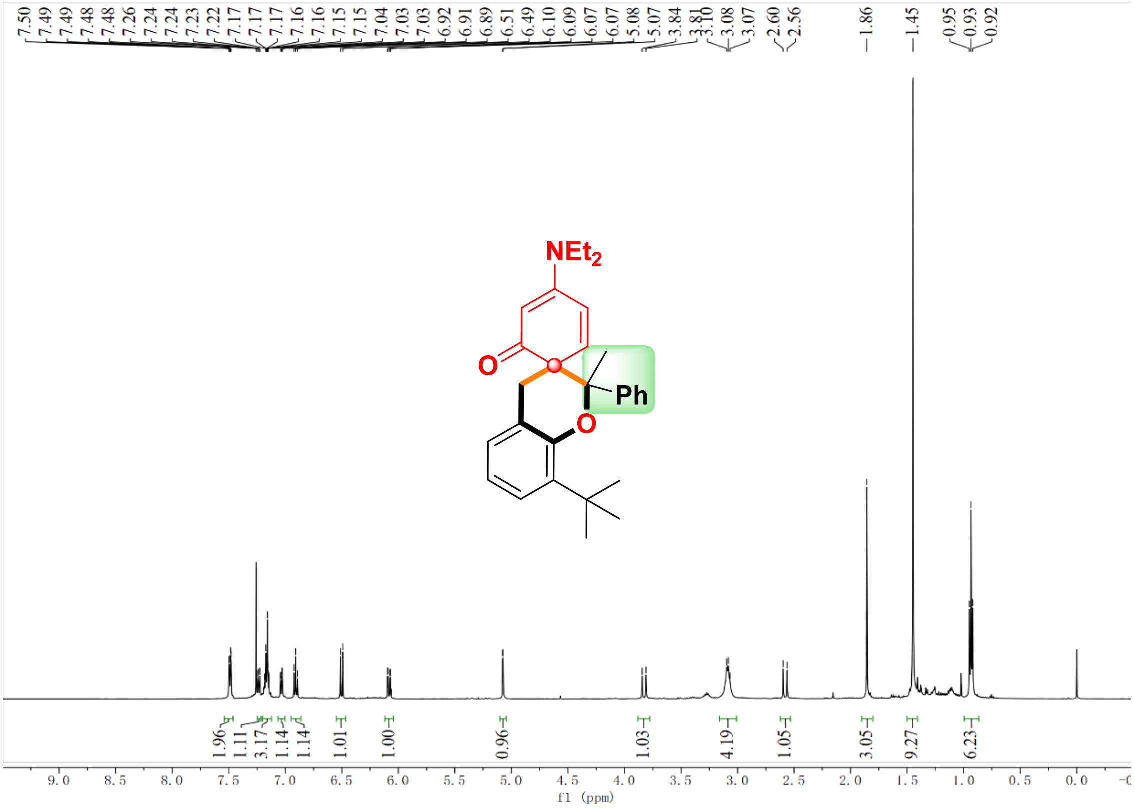

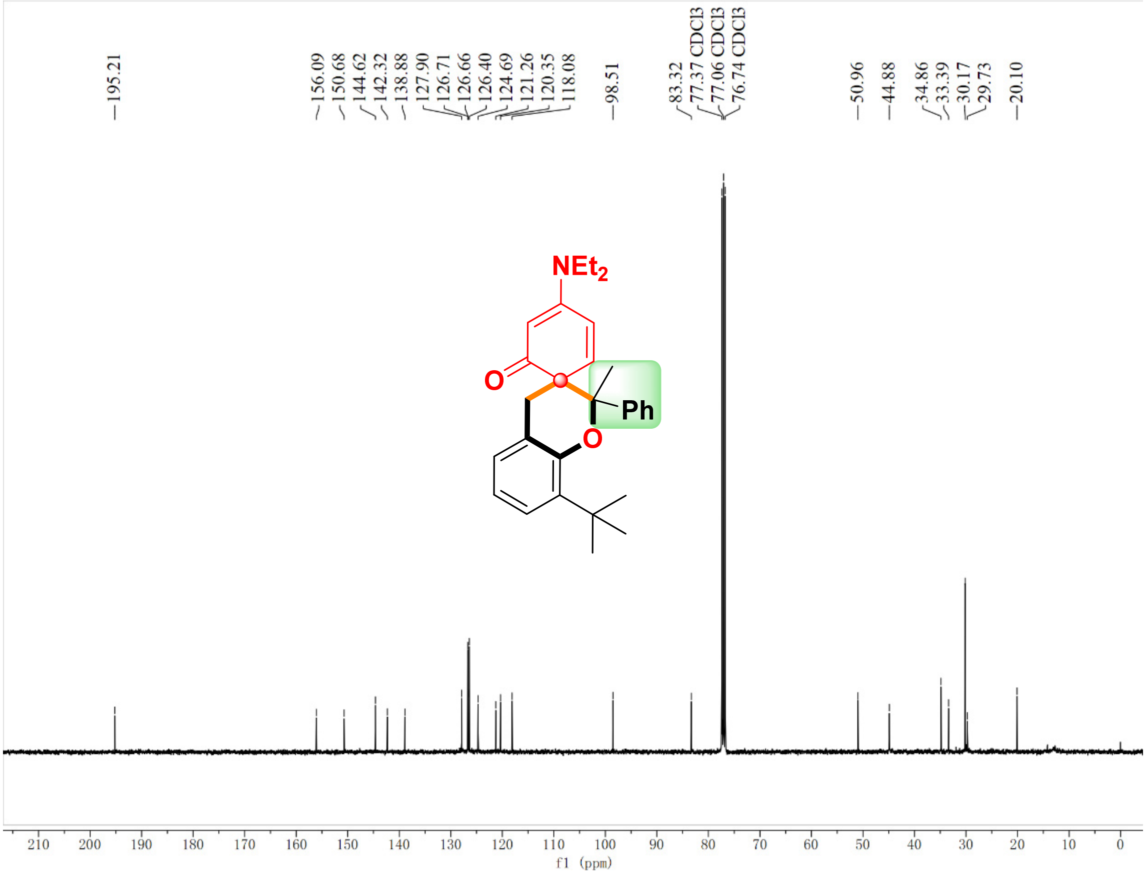

8-(tert-butyl)-4'-(diethylamino)-2-vinylspiro[chromane-3,1'-cyclohexane]-2',4'-dien-6'-one (3k)

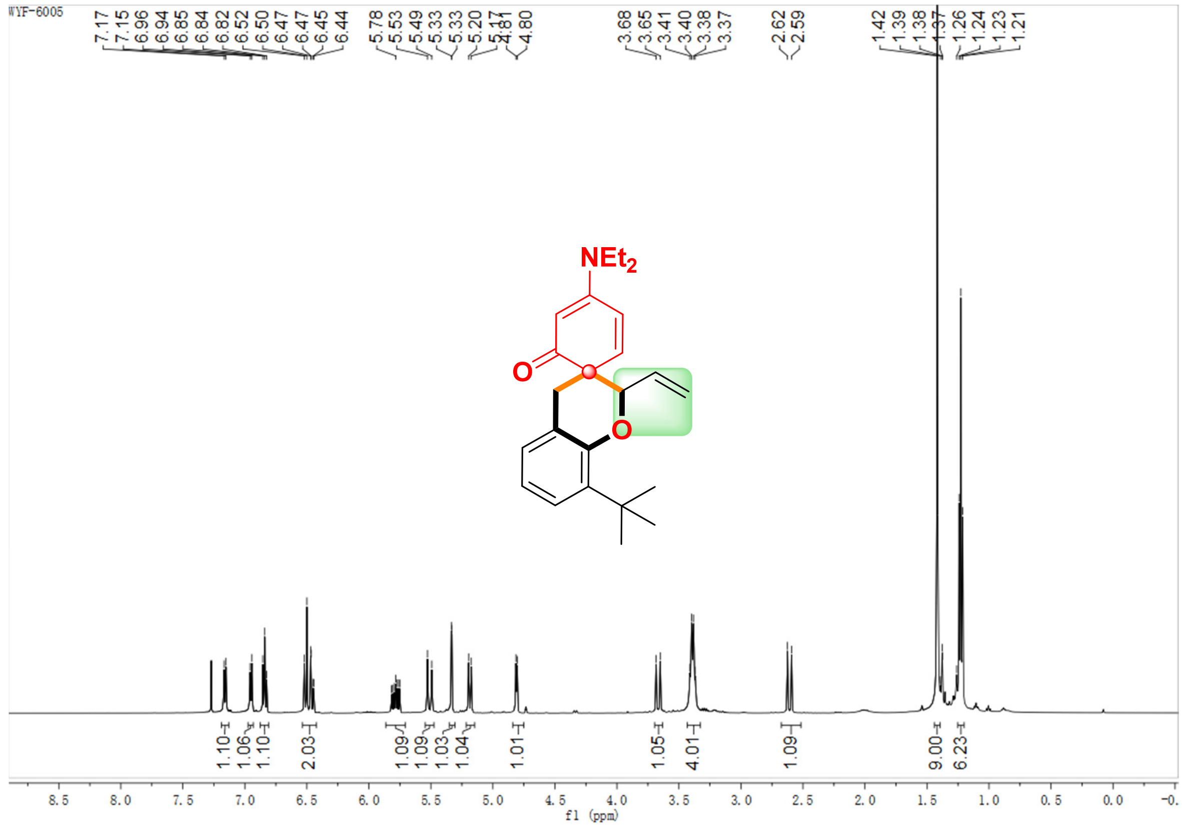

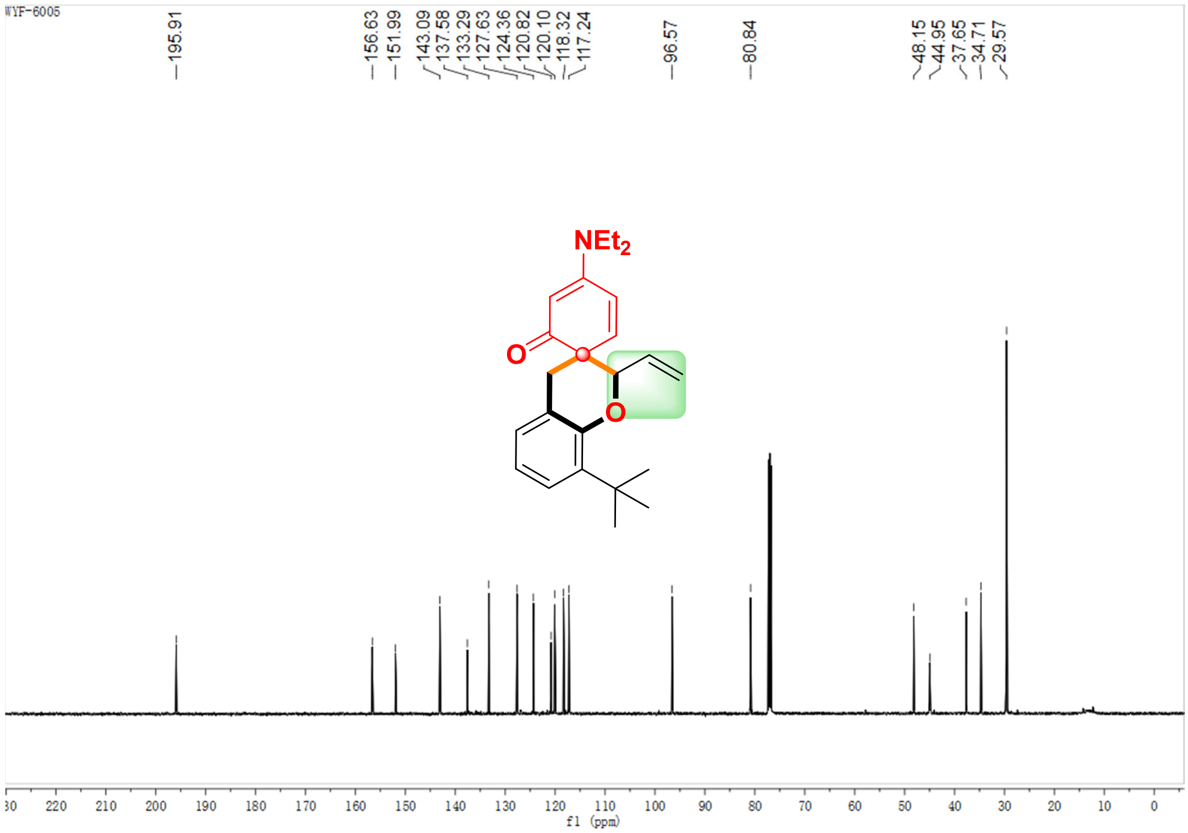

8-(tert-butyl)-4'-(dimethylamino)-2-phenylspiro[chromane-3,1'-cyclohexane]-2',4'-dien-6'-one (3l)

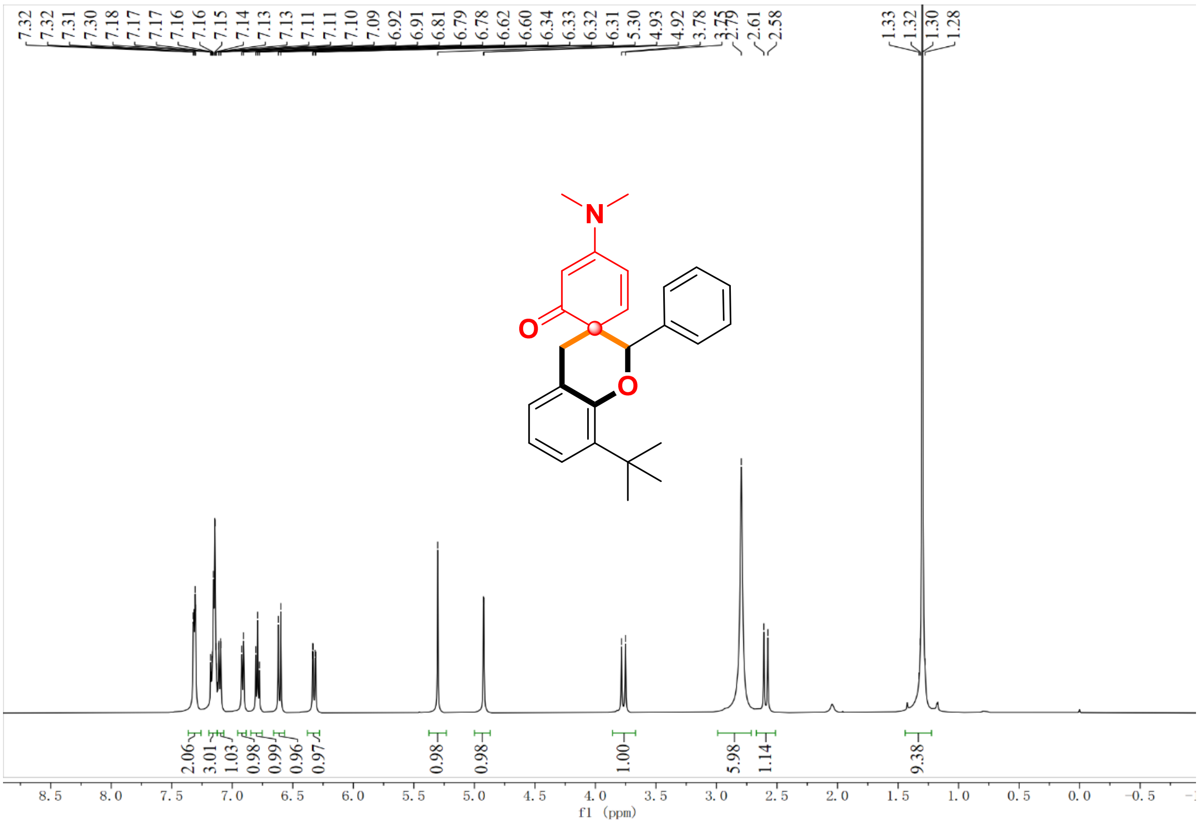

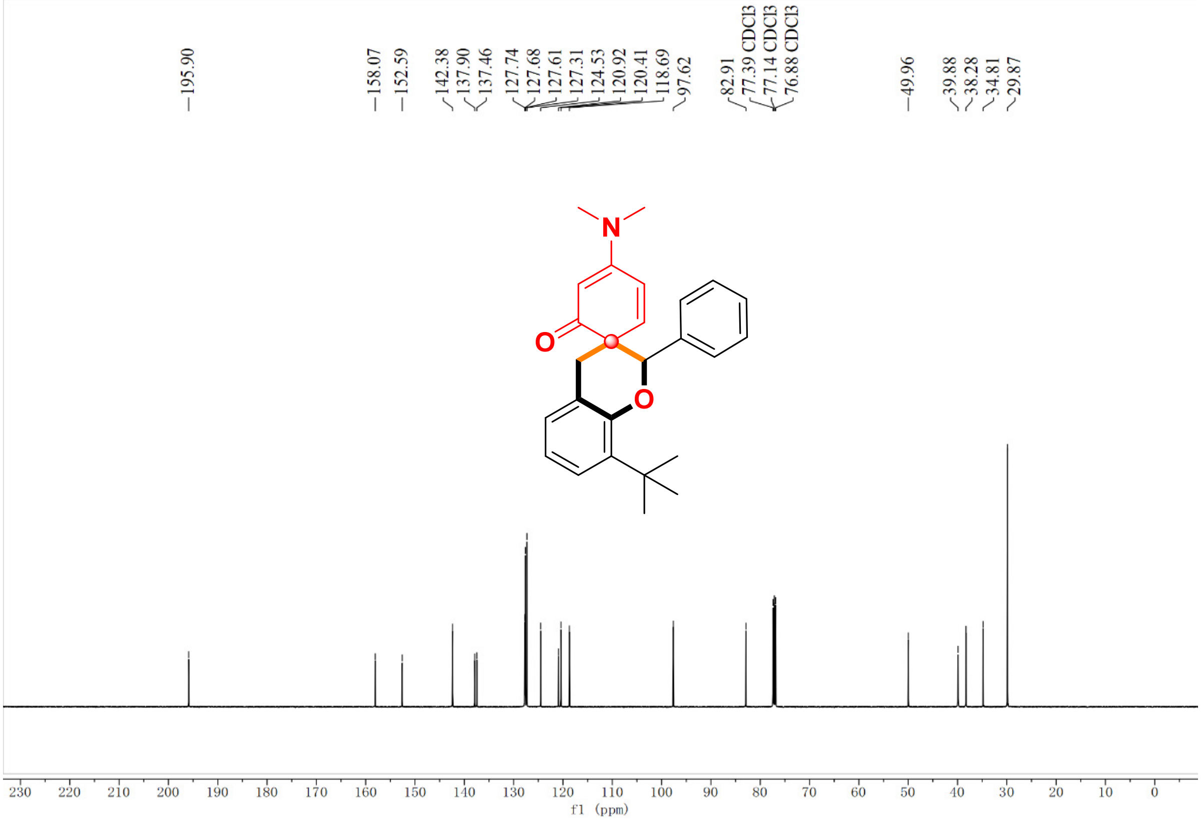

8-(tert-butyl)-2-phenyl-4'-(pyrrolidin-1-yl)spiro[chromane-3,1'-cyclohexane]-2',4'-dien-6'-one (3m)

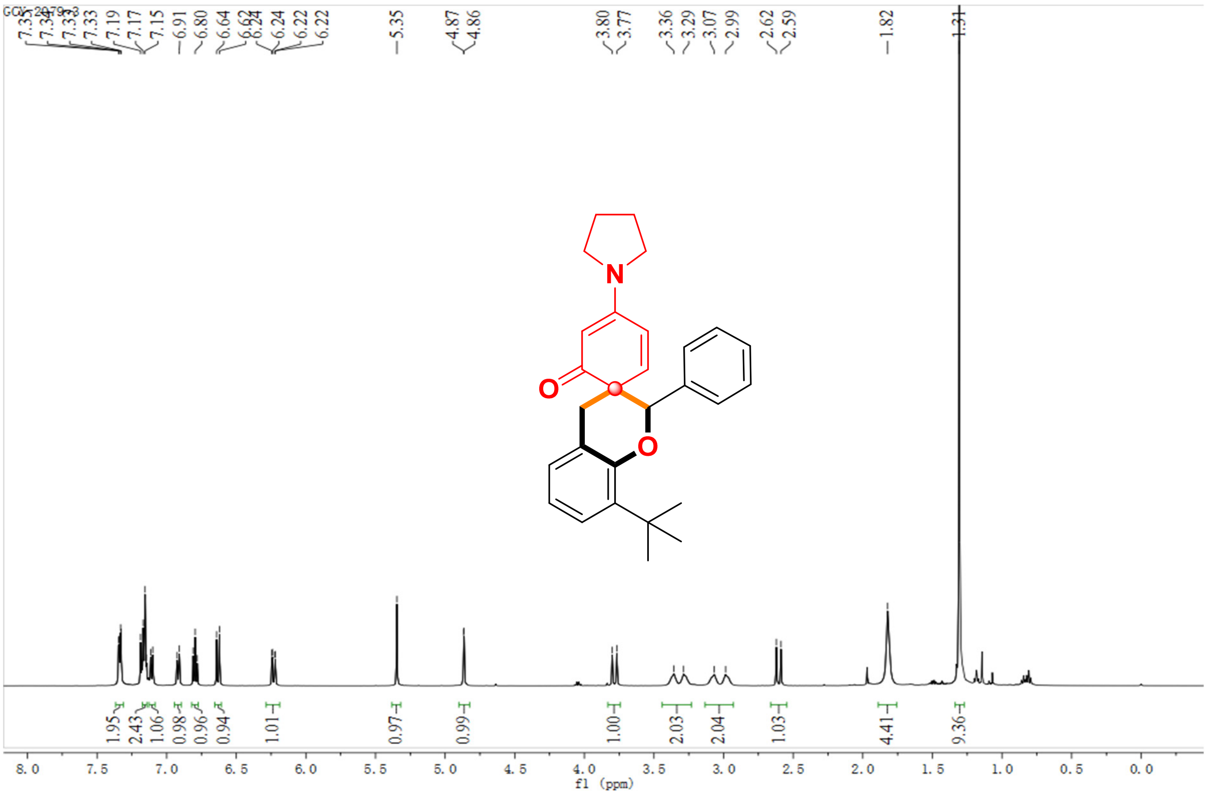

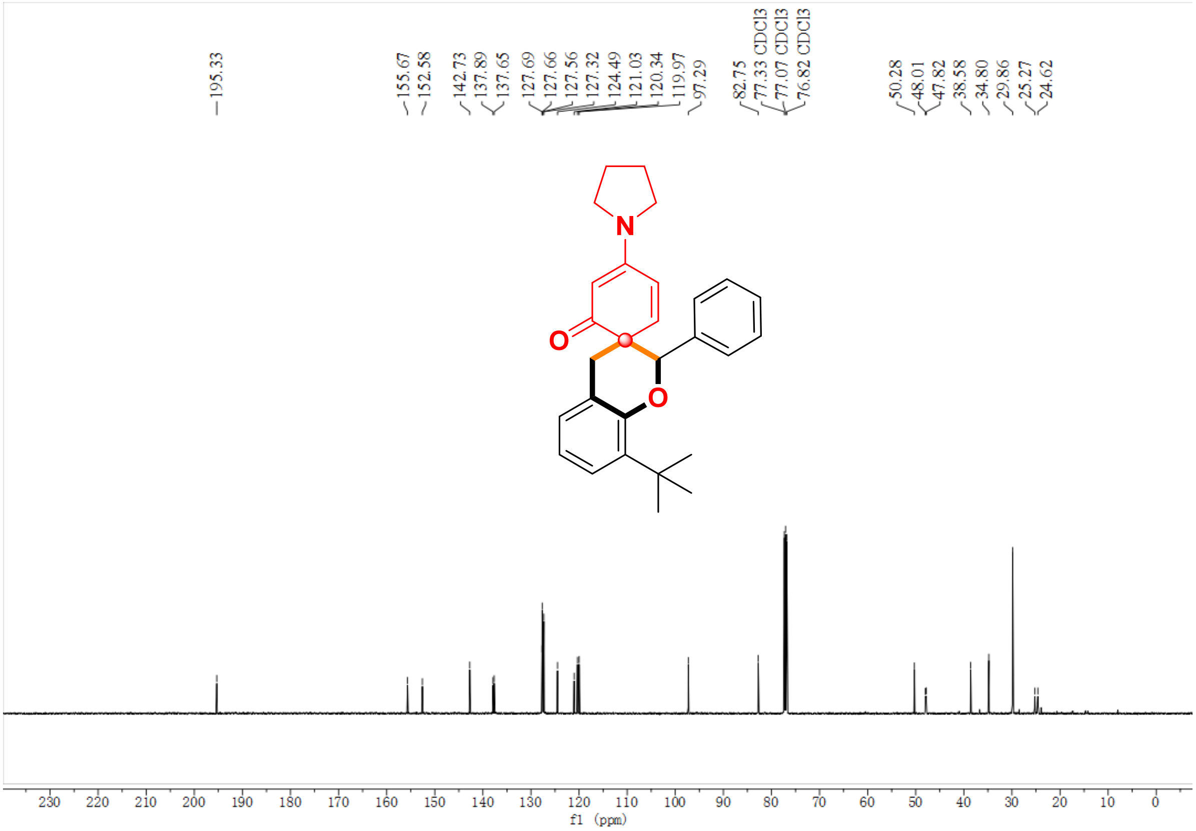

8-(tert-butyl)-2-phenyl-4'-(piperidin-1-yl)spiro[chromane-3,1'-cyclohexane]-2',4'-dien-6'-one (3n)

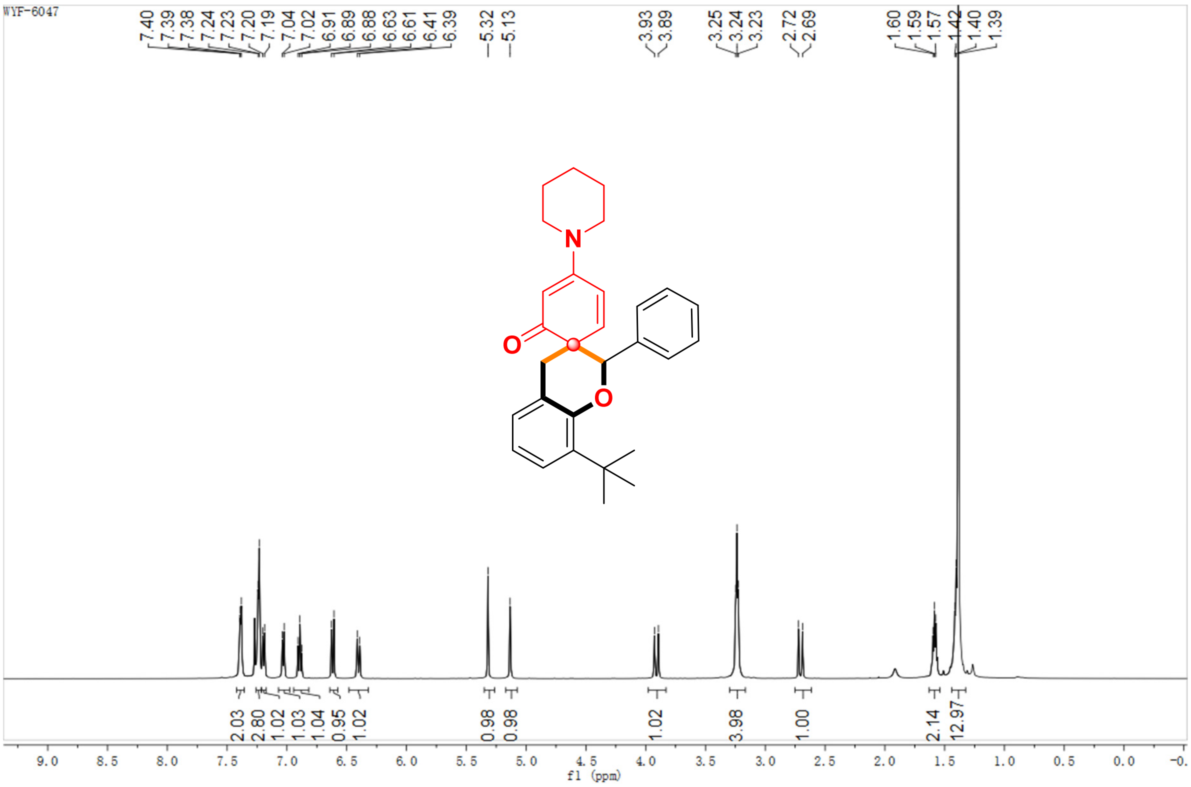

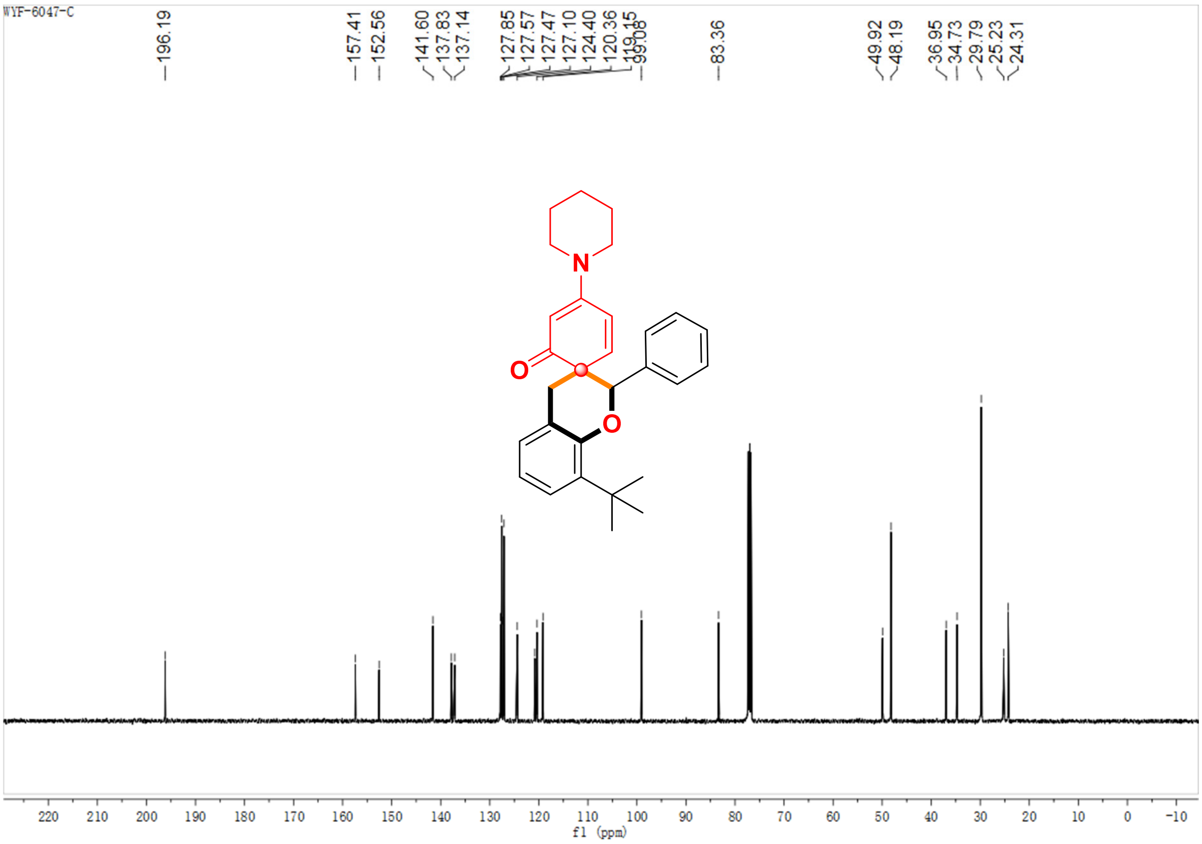

4'-(azepan-1-yl)-8-(tert-butyl)-2-phenylspiro[chromane-3,1'-cyclohexane]-2',4'-dien-6'-one (3o)

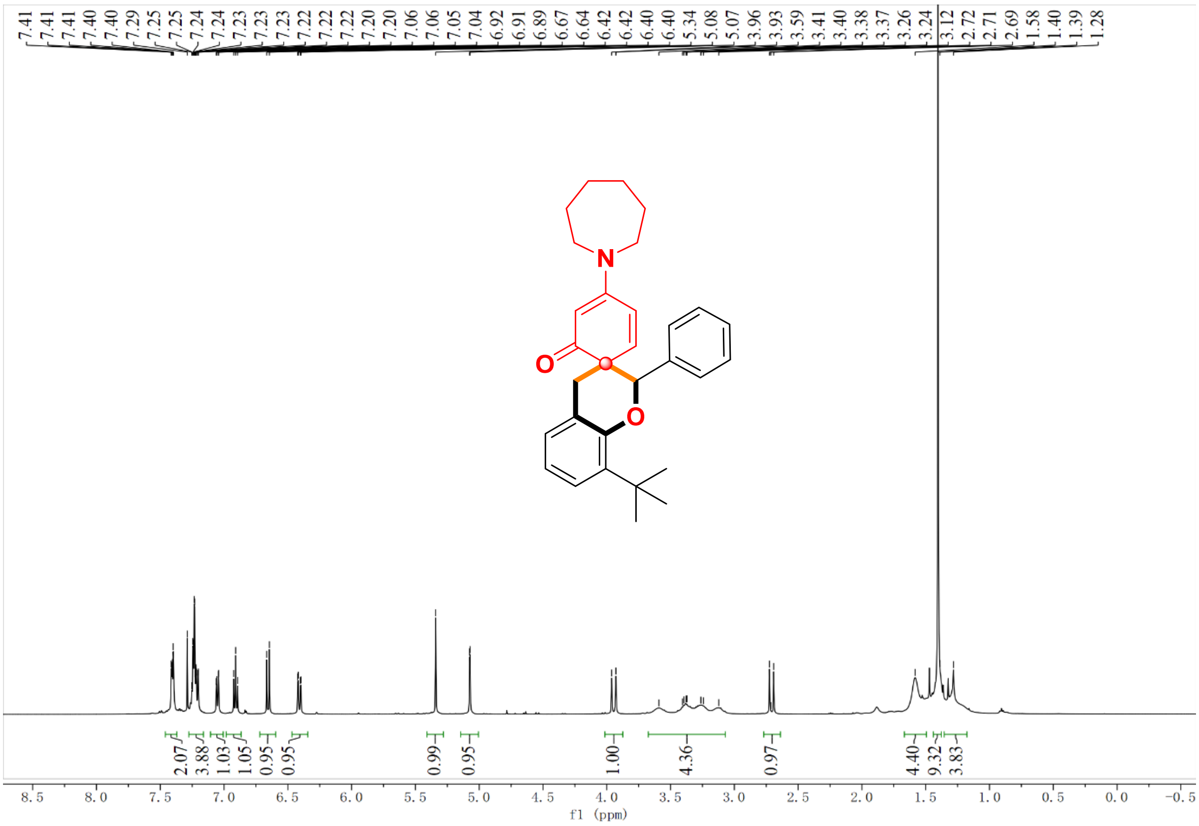

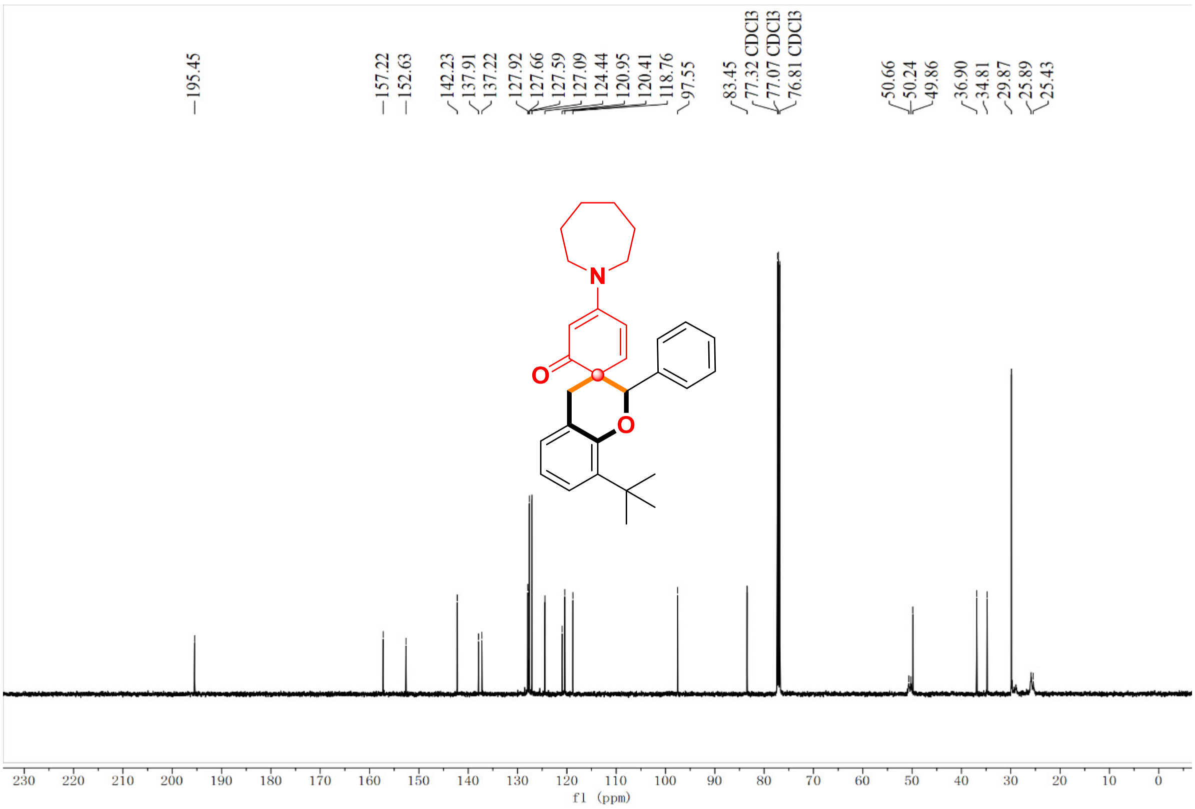

8-(tert-butyl)-4'-(dibenzylamino)-2-phenylspiro[chromane-3,1'-cyclohexane]-2',4'-dien-6'-one (3p)

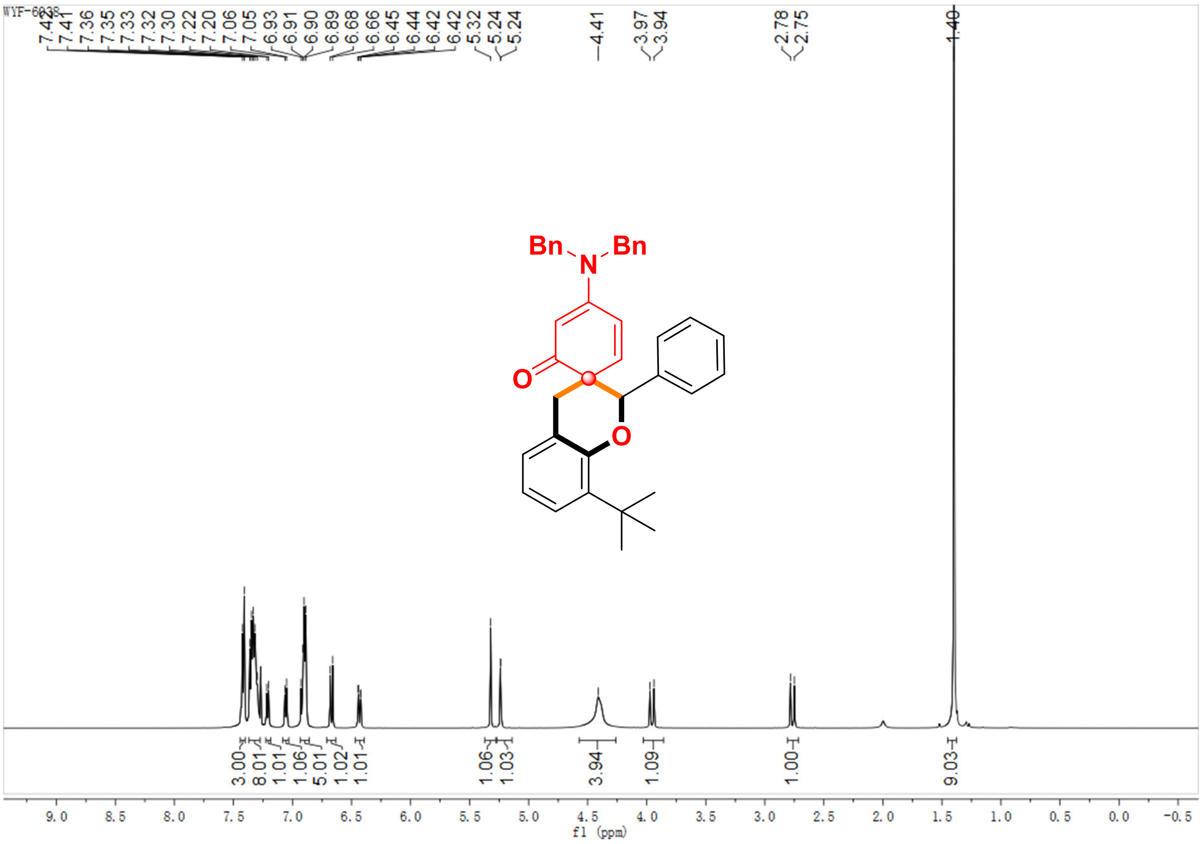

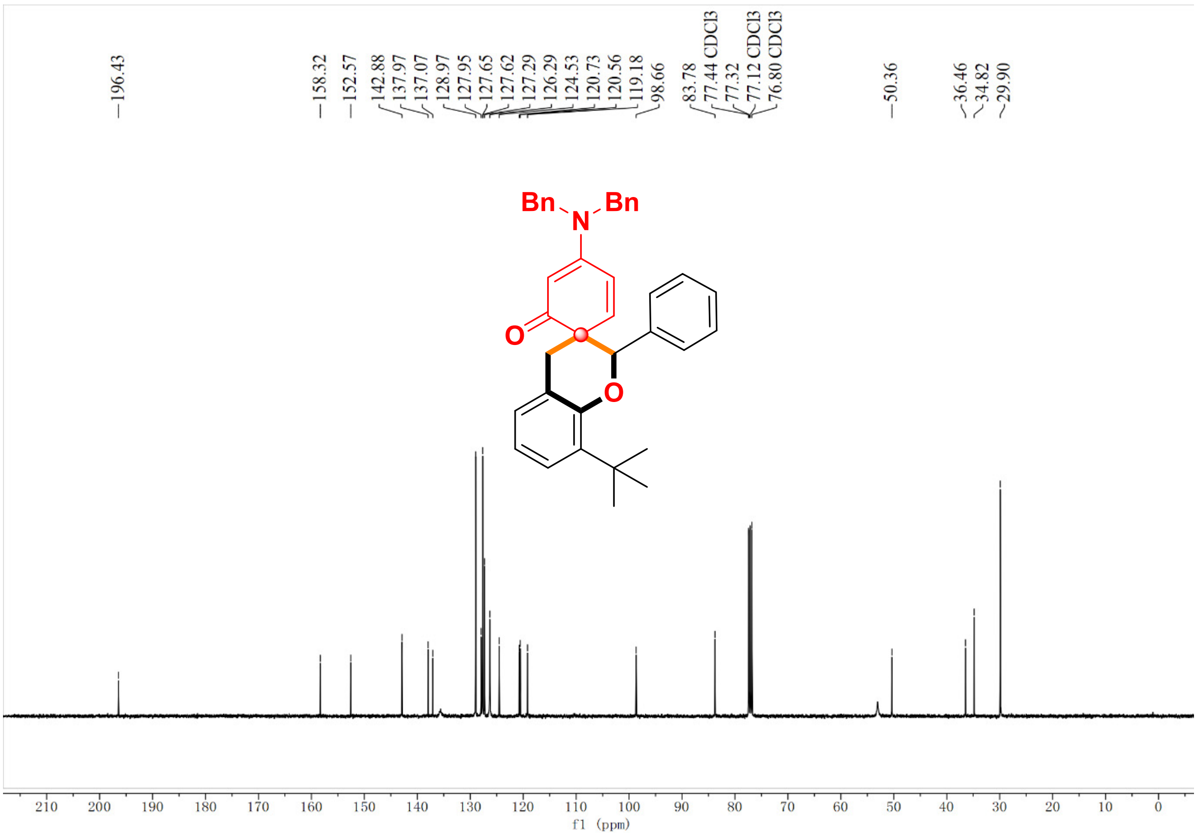

8-(tert-butyl)-4'-(diallylamino)-2-phenylspiro[chromane-3,1'-cyclohexane]-2',4'-dien-6'-one (3q)

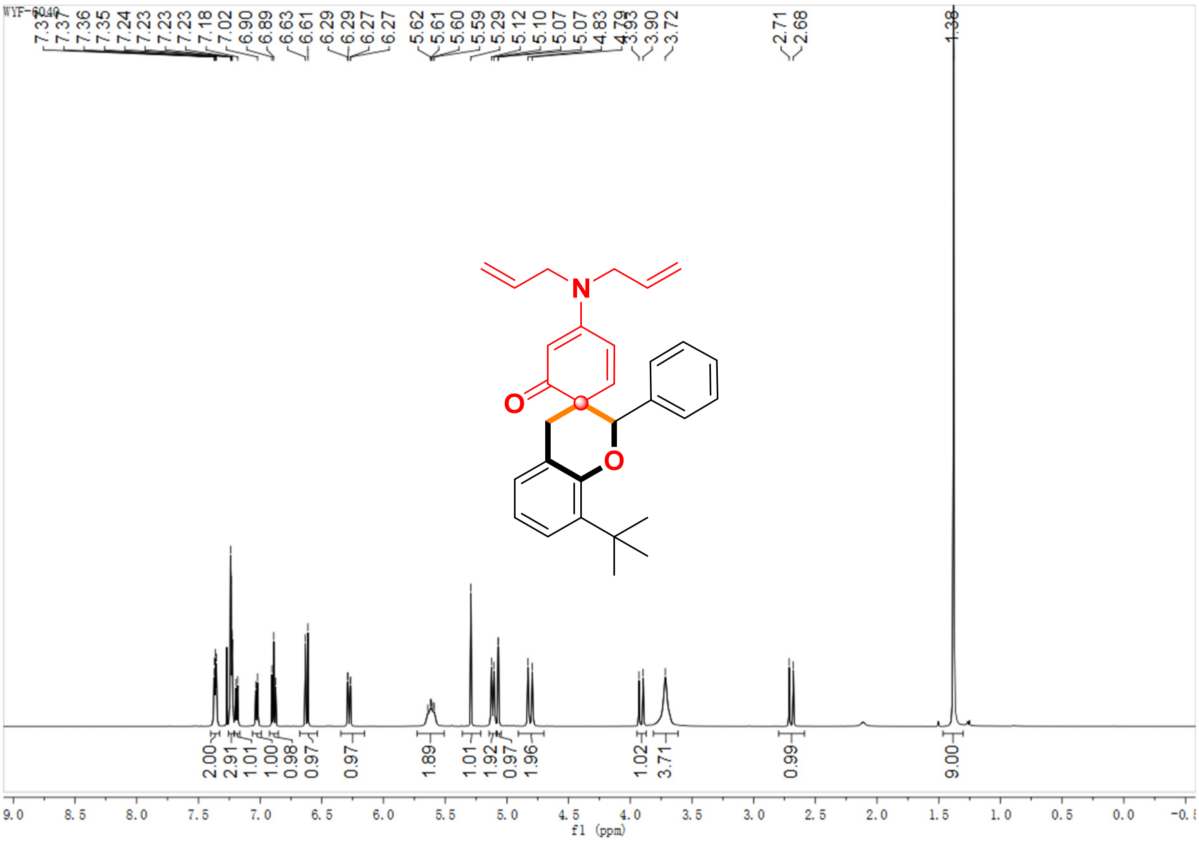

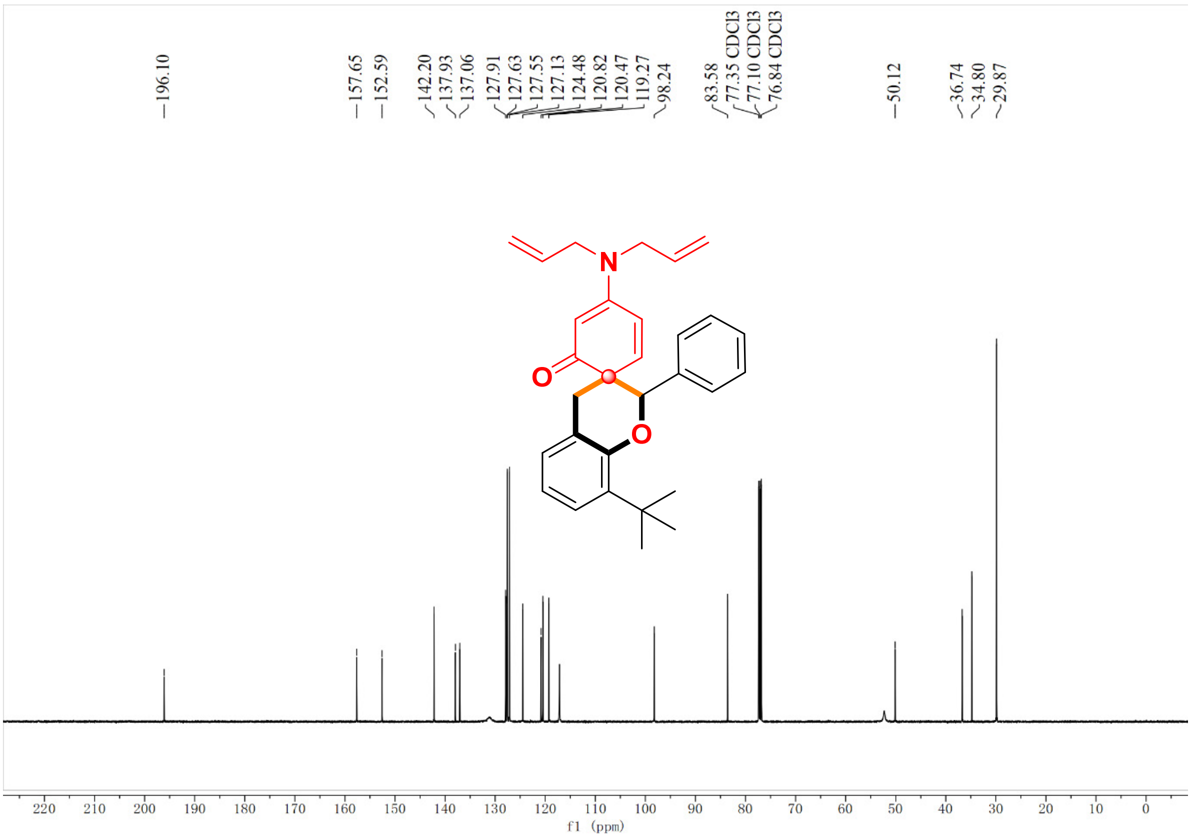

2-([1,1'-biphenyl]-4-yl)-8-(tert-butyl)-4'-(diallylamino)spiro[chromane-3,1'-cyclohexane]-2',4'-dien-6'-one (3r)

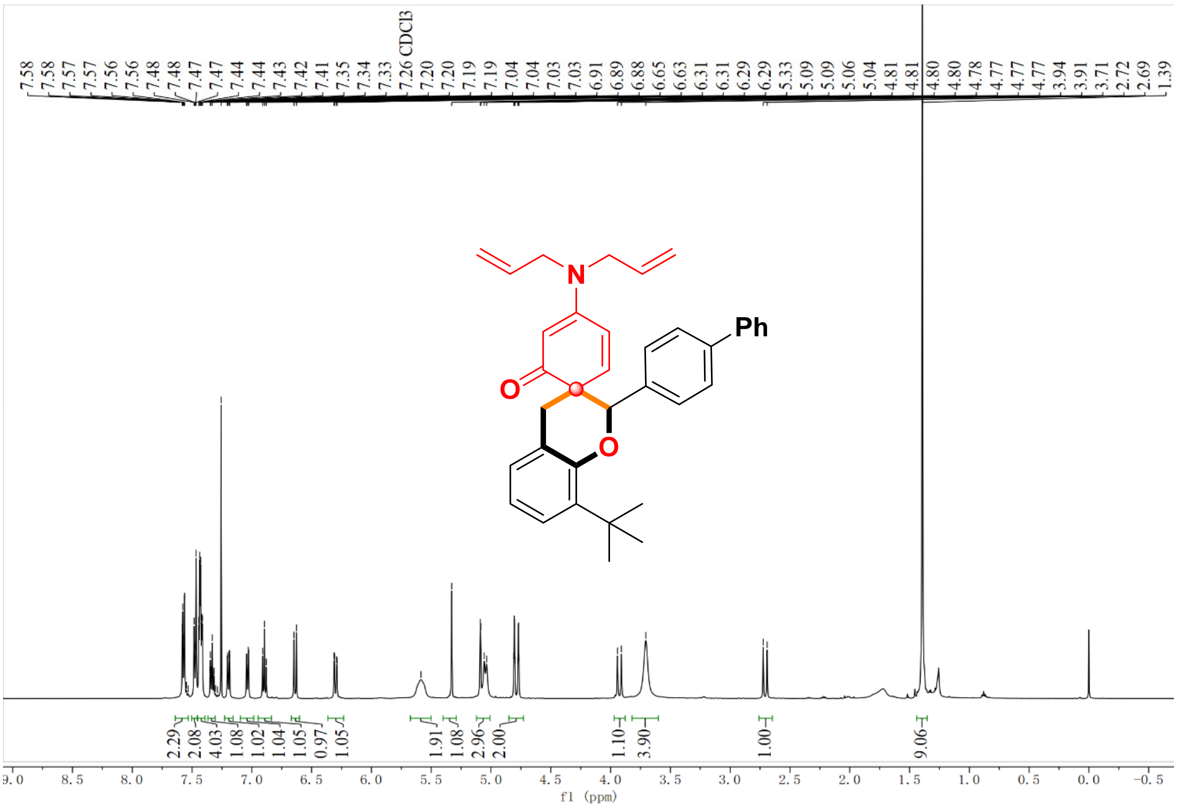

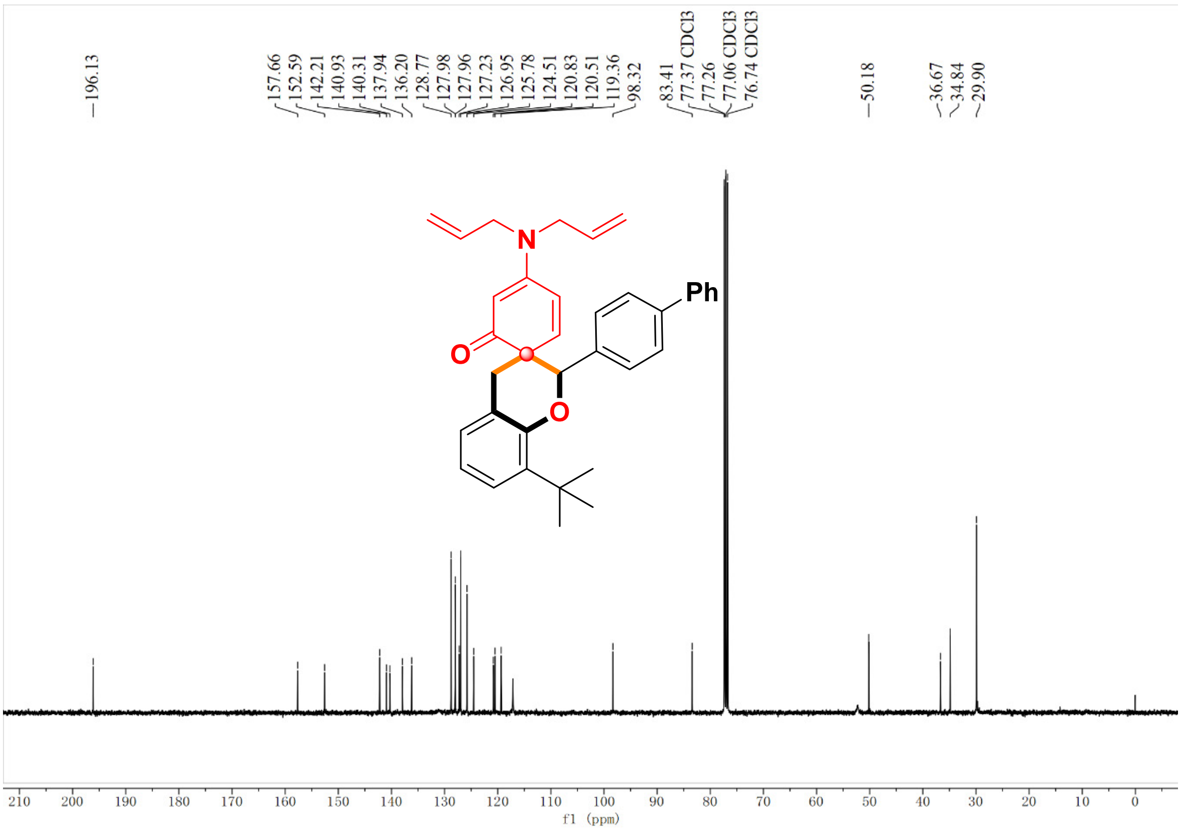

2-([1,1'-biphenyl]-4-yl)-8-(tert-butyl)-4'-(diethylamino)spiro[chromane-3,1'-cyclohexane]-2',4'-dien-6'-one (3s)

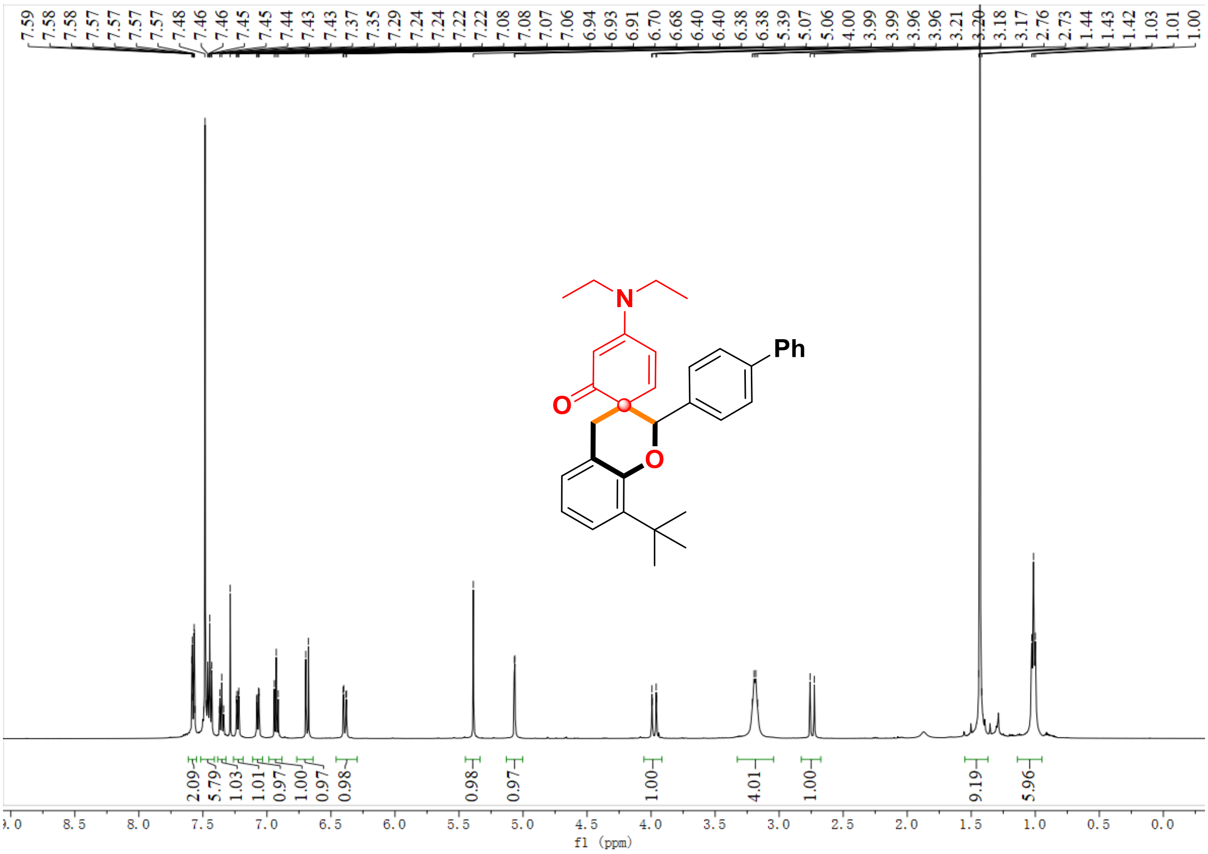

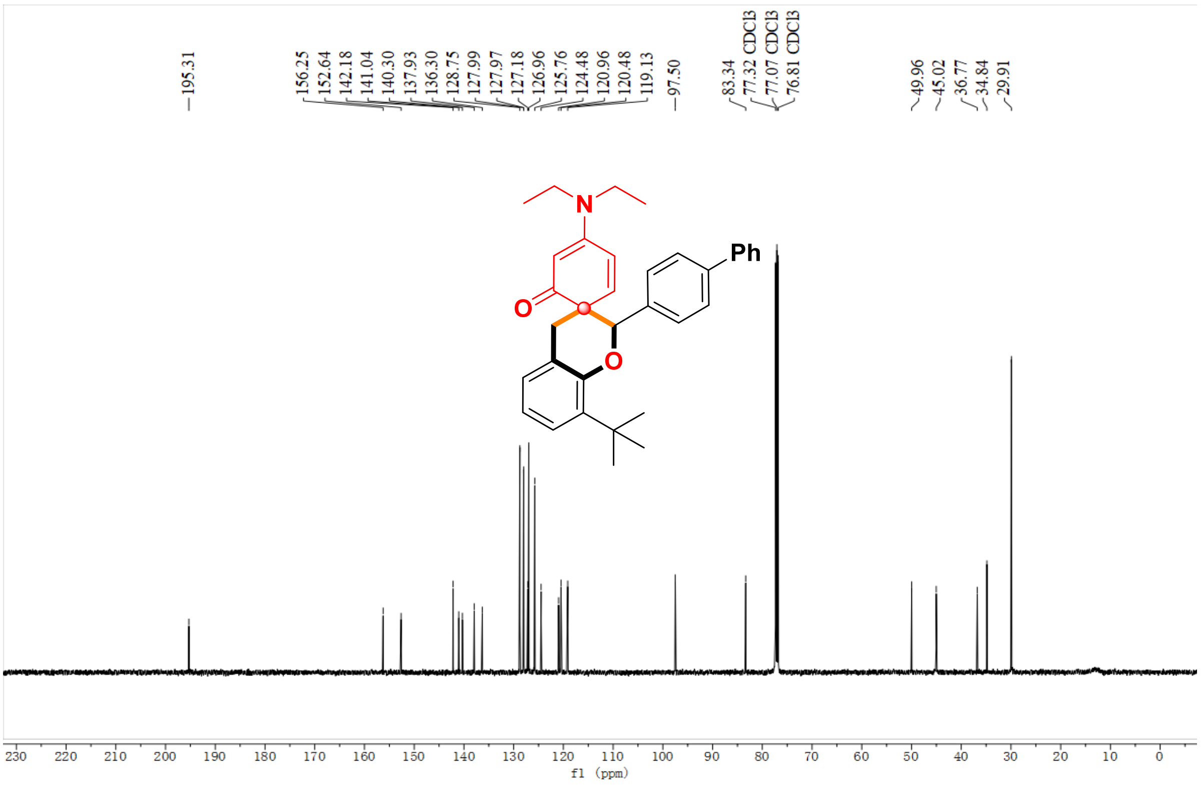

4'-(dibenzylamino)-8-isopropyl-2-phenylspiro[chromane-3,1'-cyclohexane]-2',4'-dien-6'-one (3t)

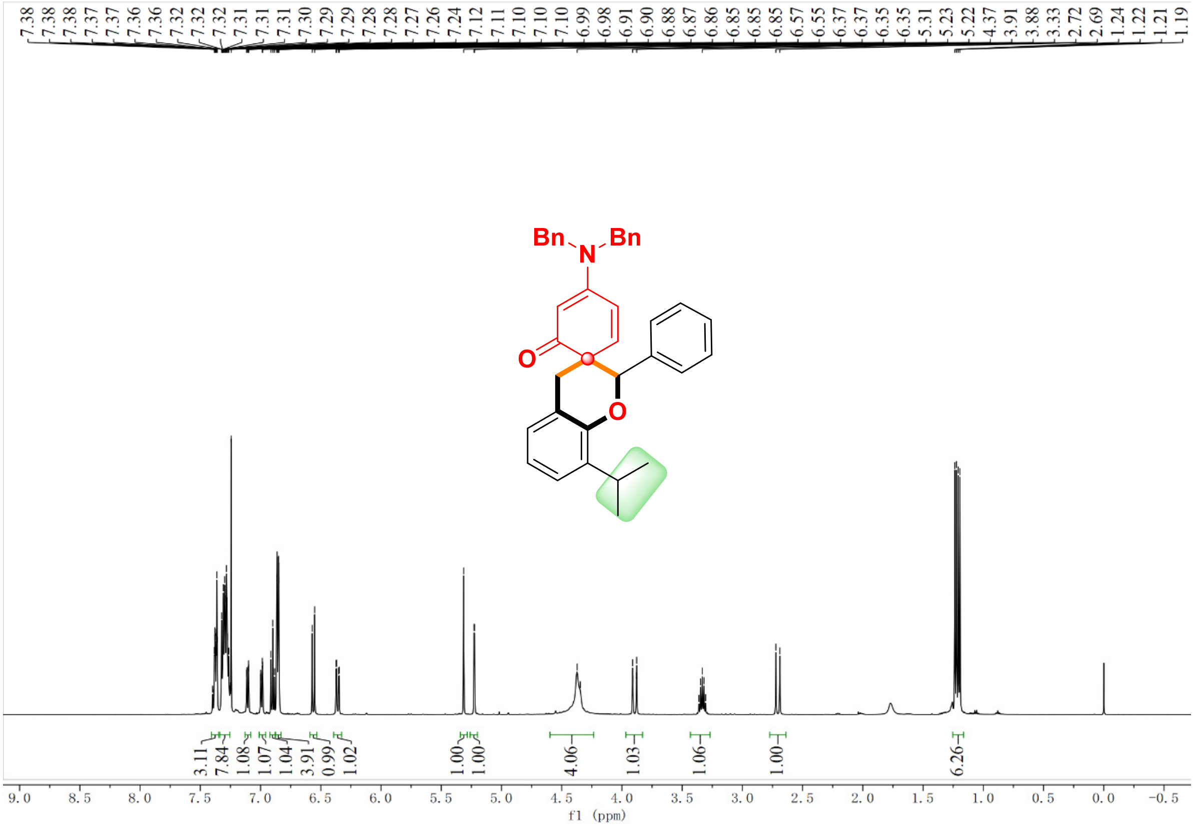

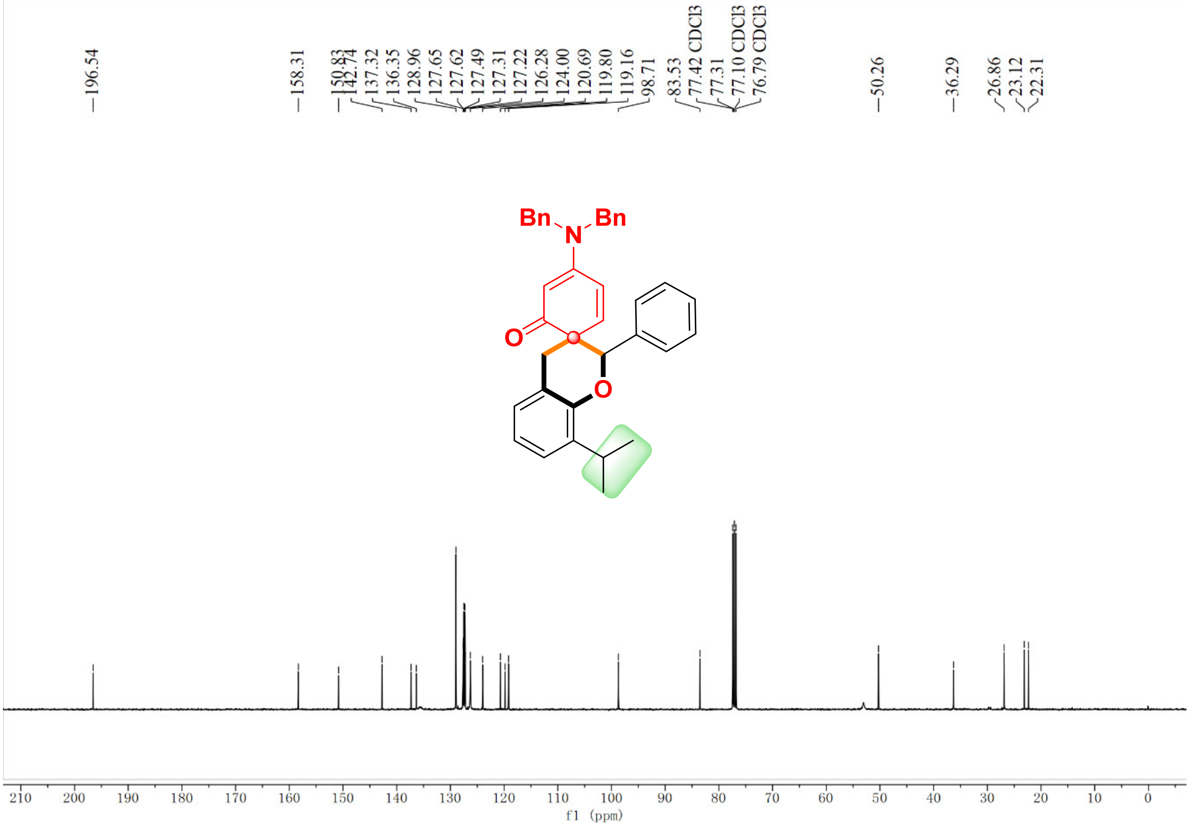

8-(sec-butyl)-4'-(dibenzylamino)-2-phenylspiro[chromane-3,1'-cyclohexane]-2',4'-dien-6'-one (3u)

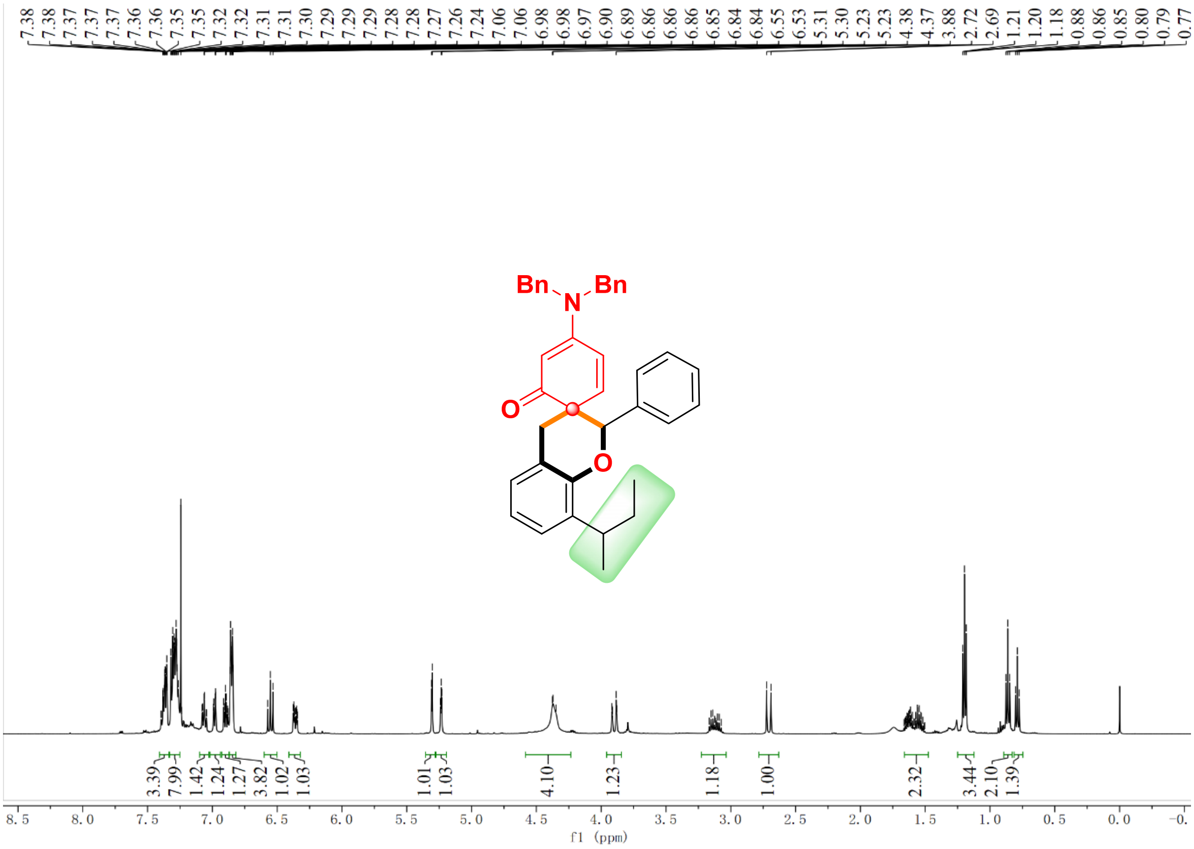

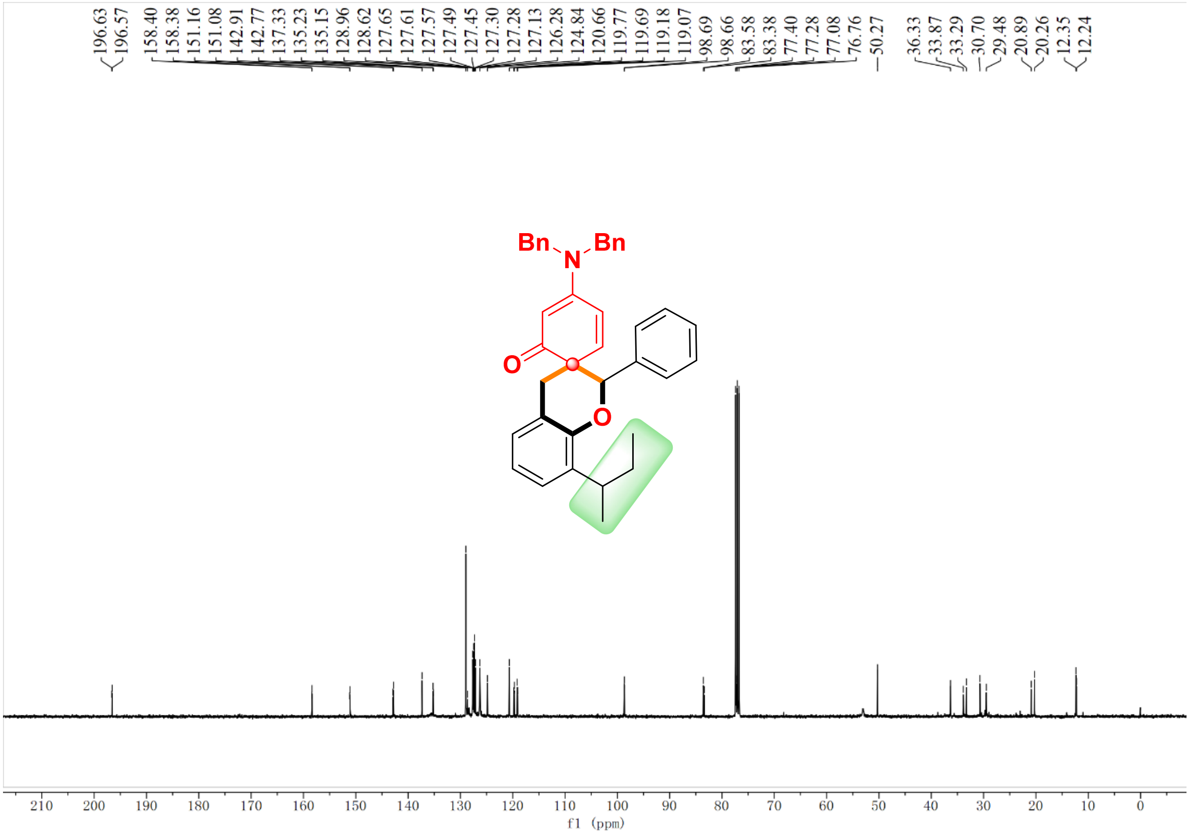

8-(tert-butyl)-4'-(diethylamino)-2-ethylspiro[chromane-3,1'-cyclohexane]-2',4'-dien-6'-one (4k)

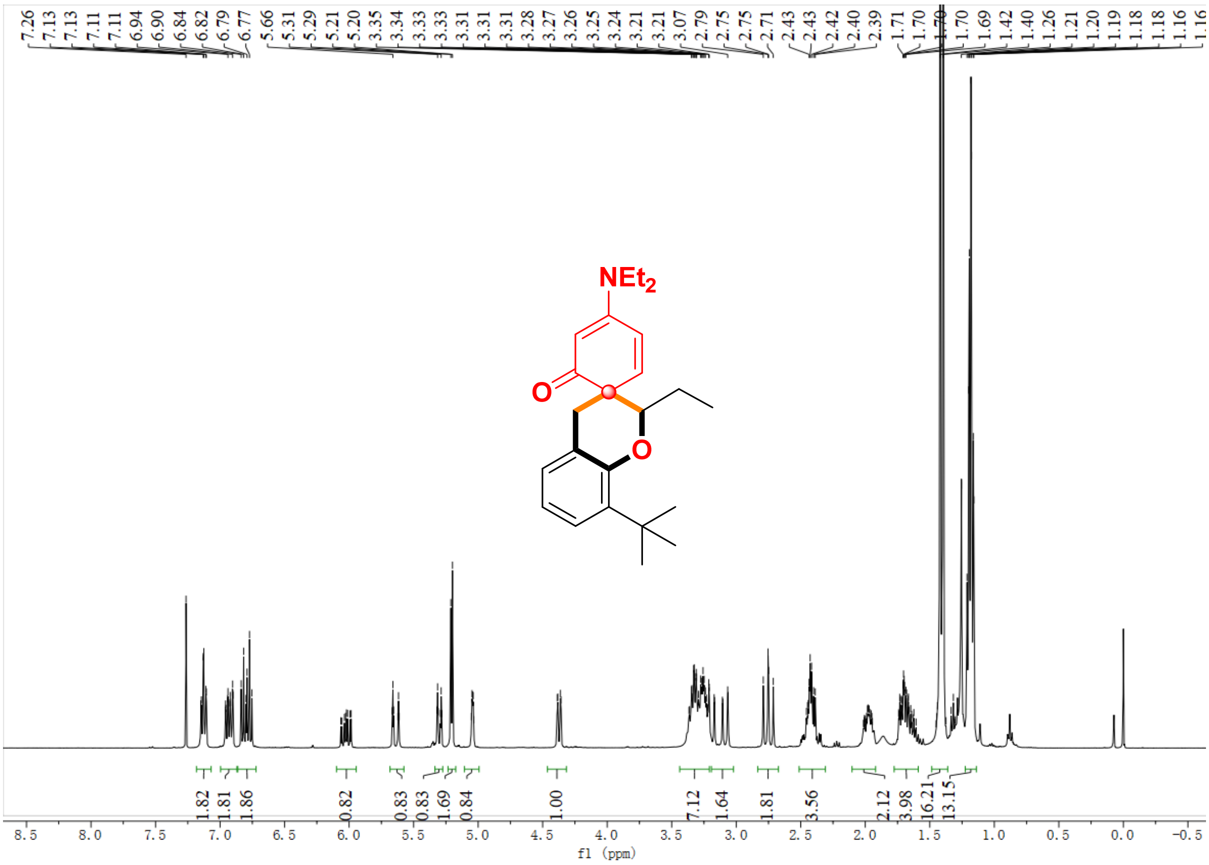

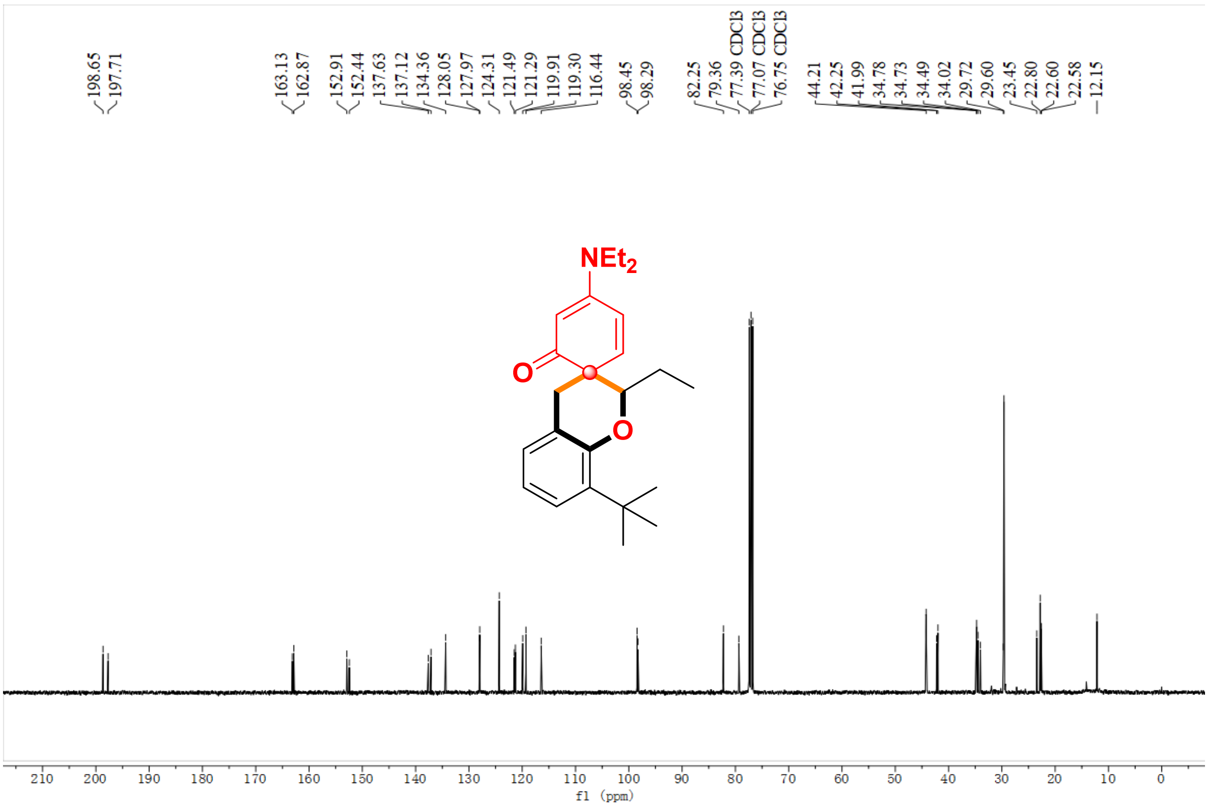

6,6'-((3-(tert-butyl)-2-((3,5-dimethoxybenzyl)oxy)phenyl)methylene)bis(3-(diethylamino)phenol) (5a)

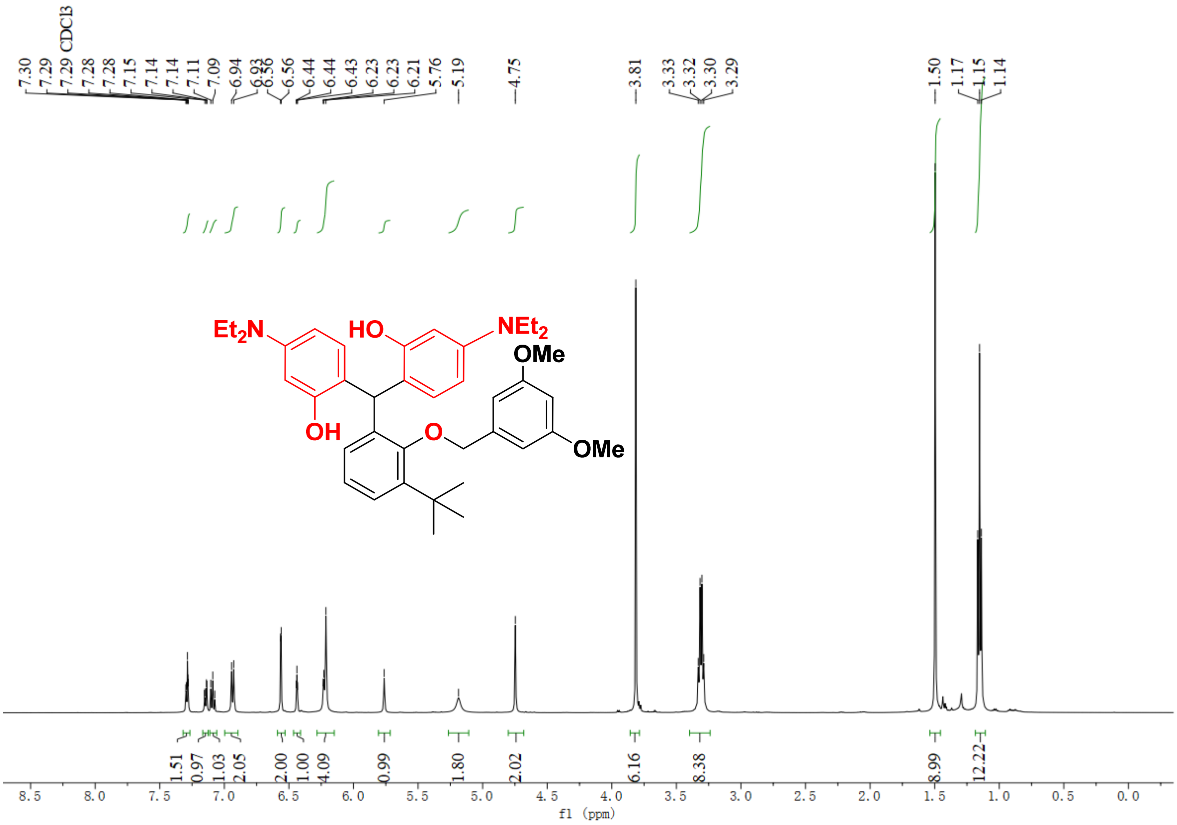

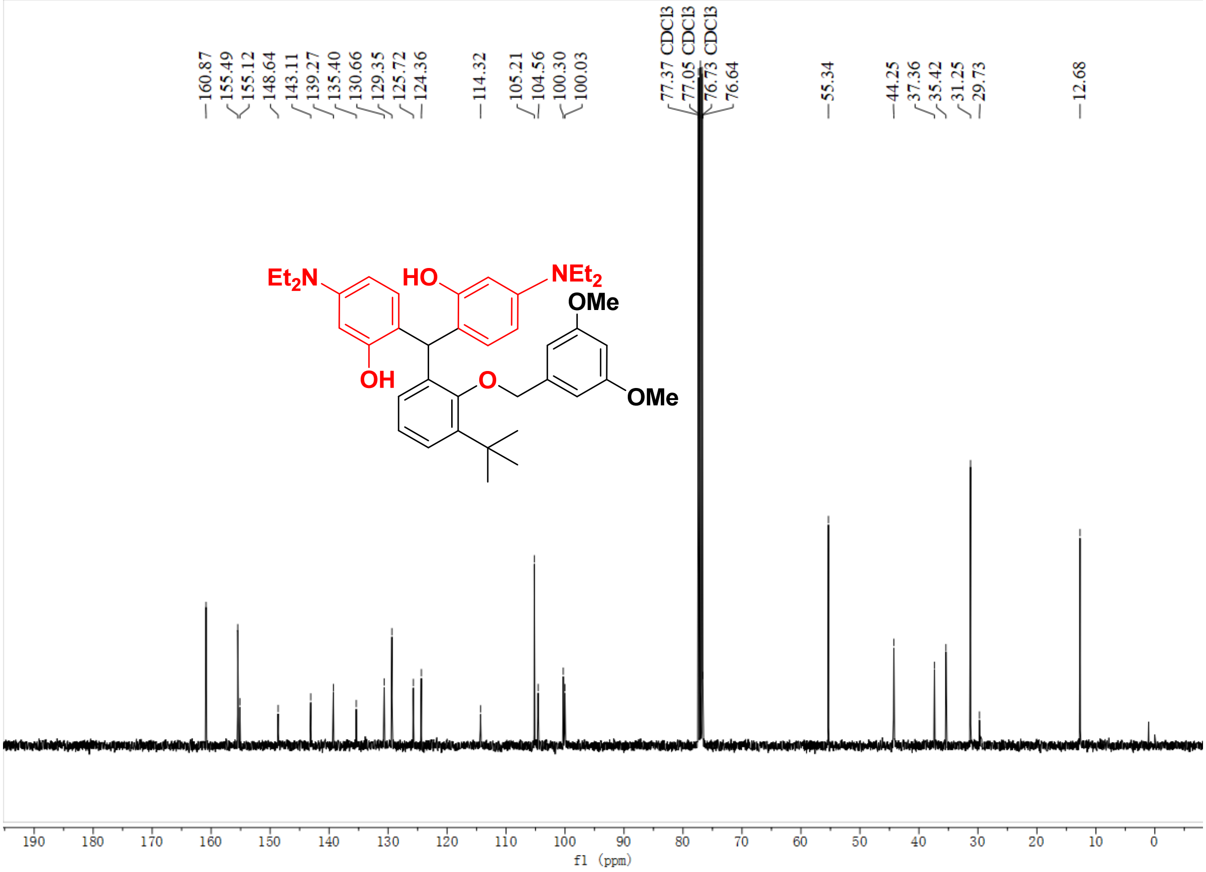

Supplement: Supplementary file 1 [file molecules-29-01012-s001.zip › molecules-2866037-supplementary.pdf]
